# Fibroblast growth factor-21 prevents diabetic cardiomyopathy via AMPK-mediated antioxidation and lipid-lowering effects in the heart

**DOI:** 10.1038/s41419-018-0307-5

**Published:** 2018-02-14

**Authors:** Hong Yang, Anyun Feng, Sundong Lin, Lechu Yu, Xiufei Lin, Xiaoqing Yan, Xuemian Lu, Chi Zhang

**Affiliations:** 1 Ruian Center of Chinese-American Research Institute for Diabetic Complications, The Third Affiliated Hospital of Wenzhou Medical University, Wenzhou, China; 2 Chinese-American Research Institute for Diabetic Complications, Wenzhou Medical University, Wenzhou, China; 3 School of Pharmaceutical Science, Wenzhou Medical University, Wenzhou, China; 4 Wenzhou Biomedical Innovation Center, Wenzhou, China

## Abstract

Our previous studies showed that both exogenous and endogenous FGF21 inhibited cardiac apoptosis at the early stage of type 1 diabetes. Whether FGF21 induces preventive effect on type 2 diabetes-induced cardiomyopathy was investigated in the present study. High-fat-diet/streptozotocin-induced type 2 diabetes was established in both wild-type (WT) and FGF21-knockout (FGF21-KO) mice followed by treating with FGF21 for 4 months. Diabetic cardiomyopathy (DCM) was diagnosed by significant cardiac dysfunction, remodeling, and cardiac lipid accumulation associated with increased apoptosis, inflammation, and oxidative stress, which was aggravated in FGF21-KO mice. However, the cardiac damage above was prevented by administration of FGF21. Further studies demonstrated that the metabolic regulating effect of FGF21 is not enough, contributing to FGF21-induced significant cardiac protection under diabetic conditions. Therefore, other protective mechanisms must exist. The in vivo cardiac damage was mimicked in primary neonatal or adult mouse cardiomyocytes treated with HG/Pal, which was inhibited by FGF21 treatment. Knockdown of AMPKα1/2, AKT2, or NRF2 with their siRNAs revealed that FGF21 protected cardiomyocytes from HG/Pal partially via upregulating AMPK–AKT2–NRF2-mediated antioxidative pathway. Additionally, knockdown of AMPK suppressed fatty acid β-oxidation via inhibition of ACC–CPT-1 pathway. And, inhibition of fatty acid β-oxidation partially blocked FGF21-induced protection in cardiomyocytes. Further, in vitro and in vivo studies indicated that FGF21-induced cardiac protection against type 2 diabetes was mainly attributed to lipotoxicity rather than glucose toxicity. These results demonstrate that FGF21 functions physiologically and pharmacologically to prevent type 2 diabetic lipotoxicity-induced cardiomyopathy through activation of both AMPK–AKT2–NRF2-mediated antioxidative pathway and AMPK–ACC–CPT-1-mediated lipid-lowering effect in the heart.

## Introduction

Diabetic cardiomyopathy (DCM) was defined as diabetes-induced impairment in the structure and function of the myocardium that was independent of hypertension and coronary artery disease^1,2^. Oxidative stress is the initial cellular pathogenesis of DCM^1–4^, which causes cardiomyocyte injury by triggering apoptosis^5,6^. Then, the apoptotic cells were replaced by the extracellular matrix that leads to cardiac remodeling and dysfunction, and ultimately results in DCM^7–9^. Therefore, efficient suppression of oxidative stress is important to prevent DCM^5,6^.

Strong evidence indicates that fibroblast growth factor (FGF)21 protects the heart from lipopolysaccharide-induced inflammation, isoproterenol-induced cardiac hypertrophy (CH), or ischemia–reperfusion-induced cardiac injury via activating antioxidative effects^10,11^. Our previous study demonstrated that FGF21 prevented the early cardiac damage in type 1 diabetic mice (T1DM) attributed to the inhibition of lipotoxicity-induced cardiac cell apoptosis^12^. Additionally, FGF21 deficiency enhanced T1DM-induced oxidative stress in the heart^13^. Since oxidative stress is the initial pathogenesis of cardiac cell apoptosis, therefore, whether FGF21 can prevent the cardiac cell apoptosis and the subsequent DCM with the mechanism of antioxidation is still unclear.

Nuclear factor (erythroid-derived 2)-like 2 (NRF2) is a master regulator of cellular detoxification responses and redox status by induction of multiple antioxidant genes expression^14,15^. Recent studies demonstrated that NRF2 agonist prevented T2DM-induced cardiomyopathy^16,17^. Further study indicated that garlic attenuates cardiac oxidative stress via activation of AKT/NRF2 pathway in fructose-fed diabetic rat^18^. And this pathway also mediated FGF21-induced prevention on diabetic nephropathy identified in our previous study^19,20^.

Strong evidence indicated that AMPK/AKT pathway is involved in cardiac protection^21,22^. A mechanistic study showed that FGF21-induced antiapoptotic effect in T1DM mice is attributed to the activation of AMP-activated protein kinase (AMPK) followed by inactivation of phosphatase and tensin homolog (PTEN) that negatively regulates AKT signaling^12^. Additionally, growing evidence indicated that activation of AMPK improved NRF2-mediated antioxidative effect^23–25^.

Since the phenotype and FGF21 level are different in two types of diabetes-induced cardiomyopathy^26^, it is unreasonable to predict the effect of FGF21 on T2DM-induced cardiomyopathy just based on the antiapoptotic effect of FGF21 in the hearts of mice at the early stage of T1DM. Therefore, in the present study, the effect of FGF21 on T2DM-induced cardiomyopathy was investigated. We found exogenous and endogenous FGF21-induced preventive effect on DCM with the mechanisms of activating AMPK-mediated antioxidative effect and lipid-lowering effect.

## Results

### FGF21 supplement prevents cardiac dysfunction in T2DM mice

Diet-induced-obesity mice model was established by 12-week-high-fat-diet (HFD) feeding (Fig. S1A), which displayed obvious insulin resistance (Fig. S1B−D). FGF21 deficiency enhanced the HFD-induced obesity and insulin resistance (Fig. S1A−D). Hyperglycemia was then induced by the single injection of STZ (Fig. S1E). Four months after the onset of diabetes, insulin resistance still existed (Fig. S2A−D), associated with lipid metabolic disorder (Fig. S2E&F). Meanwhile, plasma FGF21 notably increased in T2DM mice (Fig. S3). Although T2DM had no impact on diastolic and systolic BPs, it decreased the cardiac function (Table S1), indicating that diabetes-induced cardiac dysfunction is independent of hypertension. The above impaired cardiac function in T2DM mice was significantly prevented by FGF21 (Table S1).

### FGF21 supplement prevents cardiac hypertrophy, morphological abnormalities, and fibrosis

**Fig. 1:**
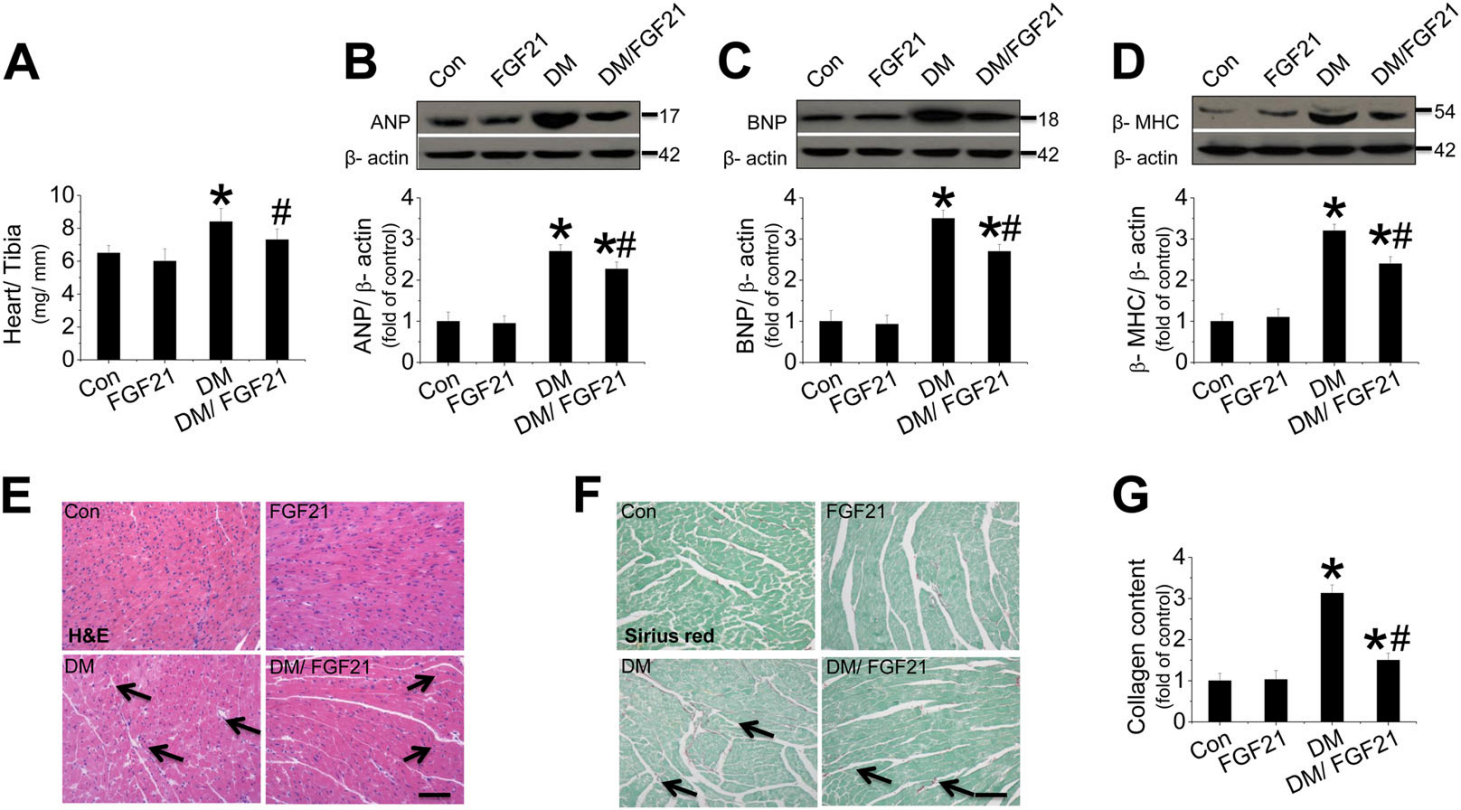
Effect of FGF21 supplement on type 2 diabetes-induced cardiomyopathy. HFD/ STZ-induced type 2 diabetic and age-matched mice received FGF21 treatment for 4 months. Then, mice were killed and the hearts were isolated. Cardiac hypertrophy was evaluated by examining the ratio of heart weight to tibia length (HW/BW, **a**) and the expression of hypertrophic markers including cardiac ANP (**b**), BNP (**c**), and β-MHC (**d**). Myocardium structure was examined by H&E staining (**e**). Fibrosis was evaluated by measuring collagen content by Sirius-red staining (**f**, **g**). Data are presented as means ± SD, *n* = 8/group. *P < 0.05 vs. the control (Con) group; ^#^*P* < 0.05 vs. the diabetic (DM) group

An obvious ratio increase of heart weight (HW) to tibia length (TL) was observed in diabetic mice compared with nondiabetic mice, which represents CH (Fig. 1a). CH was further confirmed by the increased LV mass (Table S1) and the overexpressed hypertrophic markers (Fig. 1b−d), which was prevented by FGF21 (Fig. 1b−d). Hematoxylin and eosin (H&E) and Sirius-red staining indicated that morphological abnormalities and fibrosis were observed in diabetic hearts (Fig. 1e−g), associated with overexpression of cardiac CTGF (Fig. S4A) and TGF-β (Fig. S4B). The above damage was remarkably prevented by FGF21 supplement (Fig. 1 and S4A&B). However, FGF21 only slightly decreased blood glucose level (Fig. S4C) and had no impact on plasma triglyceride (Fig. S4D), indicating that the cardiac protection of FGF21 is independent of glucose and lipid metabolic regulation.

### FGF21 prevents apoptosis and inflammation in the T2DM hearts

TUNEL assay (Fig. S5A&B) and caspase-3 cleavage (Fig. S5C&D) showed that T2DM induced apoptosis in the diabetic heart, which was strongly prevented by FGF21. Additionally, FGF21 supplement remarkably suppressed the expressions of inflammatory factors (Fig. S5E−G). We also observed that diabetes increased nuclear NF-κB p65/ I-κB ratio (Fig. S5H−J).

### FGF21 supplement prevents oxidative stress in diabetic hearts associated with improvement of NRF2 nuclear translocation

**Fig. 2:**
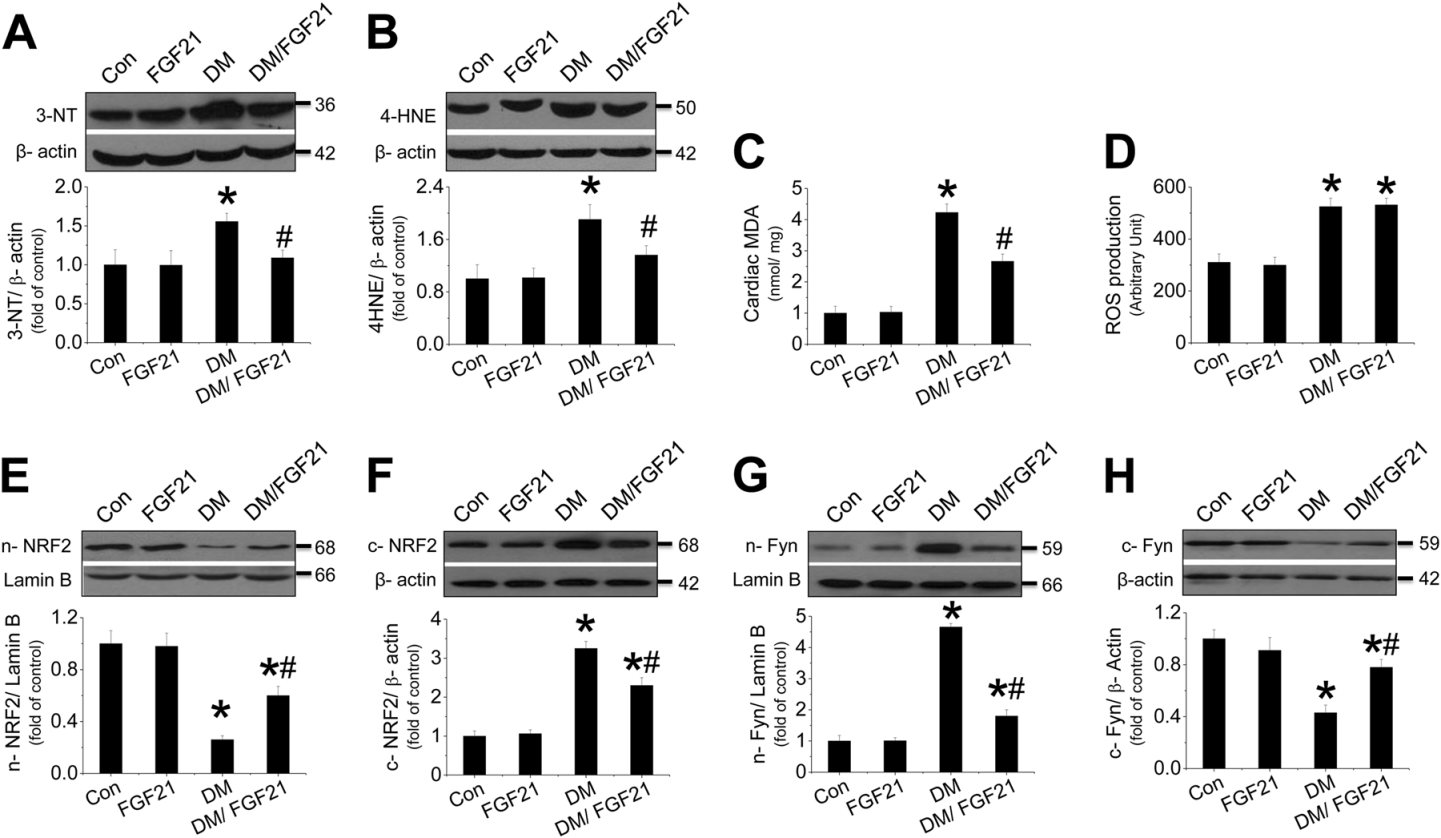
Effect of FGF21 supplement on diabetes-induced oxidative stress in the diabetic hearts. The expressions of the nitrosative damage marker 3-NT (**a**) and the oxidative marker (**b**) were measured by western blot assay. MDA (**c**) and cardiac ROS were measured (**d**) with ELISA. The translocation between the nuclei and cytosol of Nrf2 (**e**, **f**) or Fyn (**g**, **h**) was evaluated by measuring the protein of each in the nuclei and cytosol, respectively. Data are presented as means ± SD, *n* = 8/group. *P < 0.05 vs. the Con group; ^#^*P* < 0.05 vs. the DM group

FGF21 supplement suppressed T2DM-induced oxidative stress in diabetic hearts (Fig. 2a−c). Although FGF21 had no impact on diabetes-induced cardiac ROS production increase (Fig. 2d), it remarkably improved ROS clearance by induction of multiple antioxidative genes (Fig. S6A−D) in the diabetic hearts, which are the downstream target genes of NRF2. We found that T2DM inhibited NRF2 nuclear translocation (Fig. 2e, f) associated with enhanced nuclear accumulation of Fyn, a negative regulator of NRF2 (Fig. 2g, h). The impaired NRF2 nuclear translocation was reversed by FGF21 (Fig. 2e, f).

### FGF21 deficiency aggravated diabetes-induced cardiac dysfunction, hypertrophy, morphological abnormalities, and fibrosis

FGF21-KO mice were more sensitive to diabetes-induced cardiac dysfunction (Table S2). The serum insulin level (Fig. S7A), blood glucose level (Fig. S7B), and plasma triglyceride level (Fig. S7C) were increased in both WT and FGF21-KO diabetic mice. FGF21 supplement strongly improved cardiac function in both WT and FGF21-KO mice (Table S2). CH was further exacerbated in FGF21-KO mice (Fig. S7E−H). Additionally, morphological abnormalities and fibrosis in FGF21-KO diabetic hearts were more severe than in WT diabetic hearts (Fig. S7I−K). However, all the above pathological changes in FGF21-KO diabetic hearts were remarkably prevented by FGF21.

### FGF21-KO mice were sensitive to diabetes-induced cardiac apoptosis and inflammation

TUNEL staining and caspase-3 cleavage revealed that diabetes-induced cardiac apoptosis was further aggravated in FGF21-KO mice (Fig. S8A−D). However, FGF21 supplement strongly prevented cardiac apoptosis in FGF21-KO mice (Fig. S8A−D). Additionally, FGF21-KO mice were more sensitive to diabetes-induced cardiac inflammation (Fig. S8E–G), associated with the further ratio increase of NF-κB/ I-κB in the hearts (Fig. S8H−J). Anti-inflammatory effects were also observed in the hearts of FGF-KO mice treated with FGF21 (Fig. S8E−J).

### FGF21 deficiency further impairs NRF2-mediated antioxidative effect in the diabetic hearts

Compared to WT mice, FGF21-KO mice were more sensitive to diabetes-induced oxidative stress, which was strongly inhibited by FGF21 supplement (Fig. S9A−D). Meanwhile, NRF2 nuclear translocation was further suppressed in the FGF21-KO diabetic hearts (Fig. S9E, F), associated with enhanced Fyn nuclear accumulation (Fig. S9G, H). Real-time PCR assay revealed that diabetes further decreased multiple antioxidant gene expressions (Fig. S9I−L). However, the impaired nuclear translocation and function of NRF2 were rescued by FGF21 supplement (Fig. S9E−L).

### The effects of exogenous and endogenous FGF21 on HG/Pal-induced cardiomyocyte damage

Neonatal cardiomyocytes were isolated from WT and FGF21-KO mice and treated with HG/Pal. In WT cardiomyocytes, HG/Pal significantly induced apoptosis (Fig. S10A), hypertrophy (Fig. S10B), and fibrosis (Fig. S10C), all of which were further enhanced once FGF21 was deficient (Fig. S10). Moreover, a similar study was performed in adult mouse cardiomyocytes, which showed that FGF21 deficiency further enhanced HG/Pal-induced cardiomyocyte injury (Fig. S11), all of which were notably inhibited by FGF21 supplement, suggesting that the benefit of FGF21 was confirmed in adult mouse cardiomyocytes (Fig. S11).

### FGF21 induces cardiac protection against T2DM via inhibition of lipotoxicity

As we know, both glucose toxicity and lipotoxicity contribute to T2DM-induced cardiomyopathy. In order to identify which is the fighting target of FGF21, a single-factor study was performed under treatment of either HG or Pal. Although FGF21 supplement had no impact on HG-induced cardiomyocyte injury (Fig. S12), it significantly prevented Pal-induced cardiomyocyte injury (Fig. S13). Moreover, an in vivo study further confirmed that cardiomyopathy was also observed in diet-induced-obesity (DIO) mice characterized by cardiac dysfunction (Table S3), CH (Fig. S14A−D), and fibrosis (Fig. S14E, F). All the pathological changes above were remarkably prevented by FGF21 supplement (Fig. S14A−F), indicating that FGF21 prevented T2DM-induced cardiomyopathy via the inhibition of lipotoxicity rather than glucose toxicity. However, FGF21 supplement had no impact on blood glucose level and hyperlipidemia (Fig. S14G, H), suggesting that FGF21-induced cardiac protection against DIO is not the consequence of regulation of global glucose and lipid metabolism.

### AMPK mediated FGF21-induced prevention on cardiac cell injury against HG/Pal

**Fig. 3:**
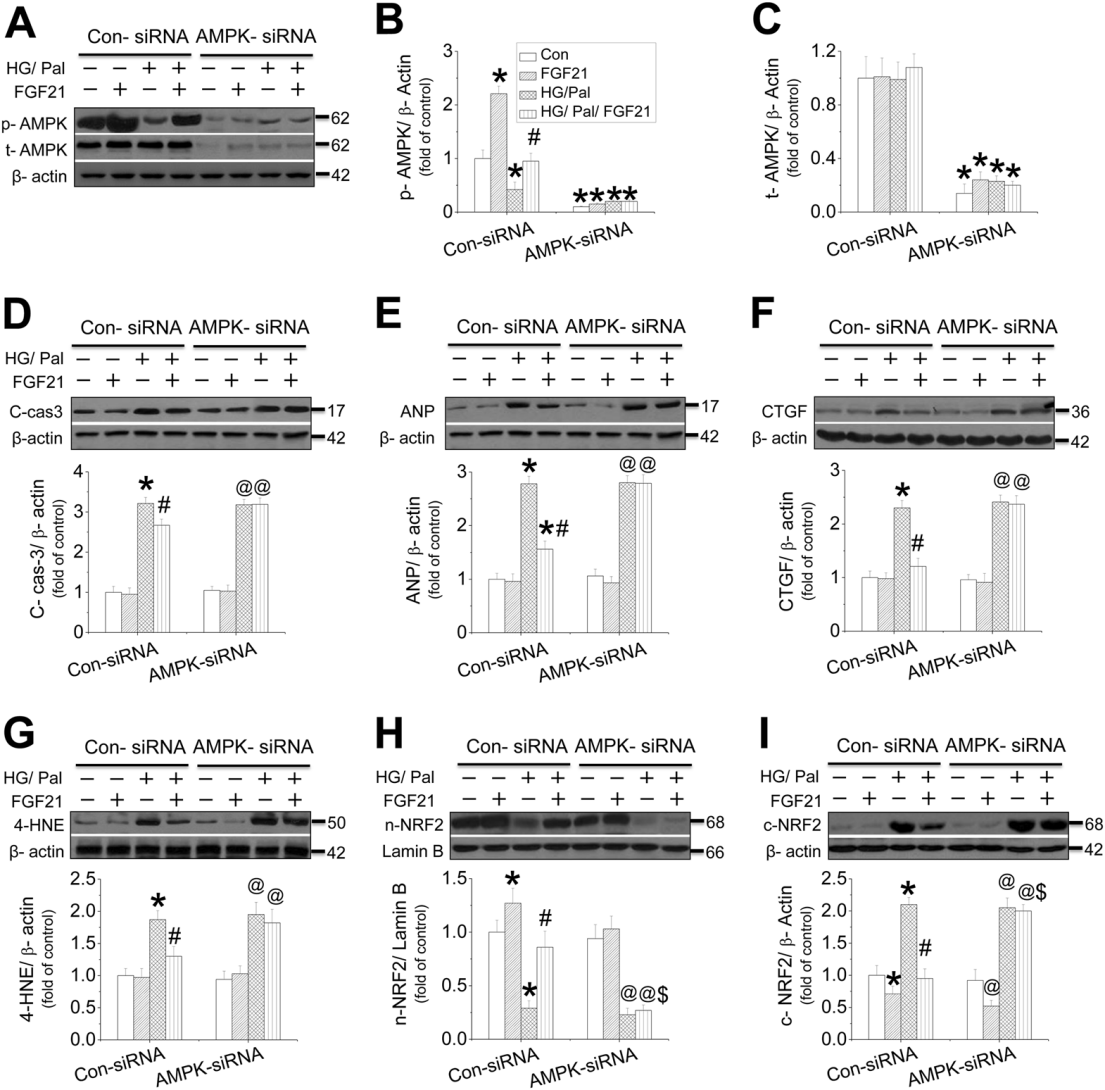
The role of AMPK in FGF21-induced protection in cardiomyocytes against HG/ Pal. Primary cardiomyocytes were isolated from neonatal mice and treated with either control or AMPK-specific siRNA and then the cells were cotreated with both HG/Pal and FGF21 for 24 h. Western blotting was used to detect the phosphorylation (**a**, **b**) and expression levels of AMPK (**a**, **c**). Under this circumstance, the expressions of cleaved-caspase 3 (**d**), ANP (**e**), CTGF (**f**), and 4-HNE (**g**) were measured by western blot assay. The translocation between the nuclei and cytosol of Nrf2 (**h**, **i**) was evaluated by measuring the protein of each in the nuclei and cytosol, respectively. Data were collected from at least three independent experiments and presented as mean ± SD. *P < 0.05 vs. control in the Con-siRNA group; ^#^*P* < 0.05 vs. HG/Pal in the Con-siRNA group; ^@^*P* < 0.05 vs. control in AMPK-siRNA group; ^$^*P* < 0.05 vs. HG/Pal/FGF21 in the Con-siRNA group

An in vivo study showed that FGF21 increased, but DM decreased cardiac AMPK phosphorylation (Fig. S15A), which was further decreased in the diabetic heart of FGF21-KO mice (Fig. S15B). However, the impaired AMPK activity in diabetic hearts was remarkably rescued by the supplement of FGF21 in both FGF21-KO mice (Fig. S15). In an in vitro mechanistic study, neonatal mouse cardiomyocytes were isolated and treated with HG/Pal in the presence of AMPK-siRNA. The results showed that inhibition of AMPK phosphorylation and expression by AMPK-siRNA (Fig. 3a−c) completely blocked FGF21-induced suppression on HG/Pal-induced apoptosis (Fig. 3d), hypertrophy (Fig. 3e), fibrosis (Fig. 3f), and oxidative stress (Fig. 3g) in cardiomyocytes. Meanwhile, AMPK-siRNA blocked FGF21-induced NRF2 nuclear translocation (Fig. 3h, i) and its downstream target genes expression associated with reduction of nuclear accumulation (Fig. S16). Similar studies were also performed in adult mouse cardiomyocytes which showed that FGF21 increased, but HG/Pal decreased AMPK phosphorylation (Fig. S17A, B). Knockdown of AMPK with AMPK-siRNA (Fig. S17A, C) blocked FGF21-induced cardiomyocyte protection (Fig. S17D−G), associated with inhibition of FGF21-induced NRF2 nuclear translocation (Fig. S17H) and AKT phosphorylation (Fig. S17I).

### NRF2 partially mediated FGF21-induced cardiomyocyte protection against HG/Pal

**Fig. 4:**
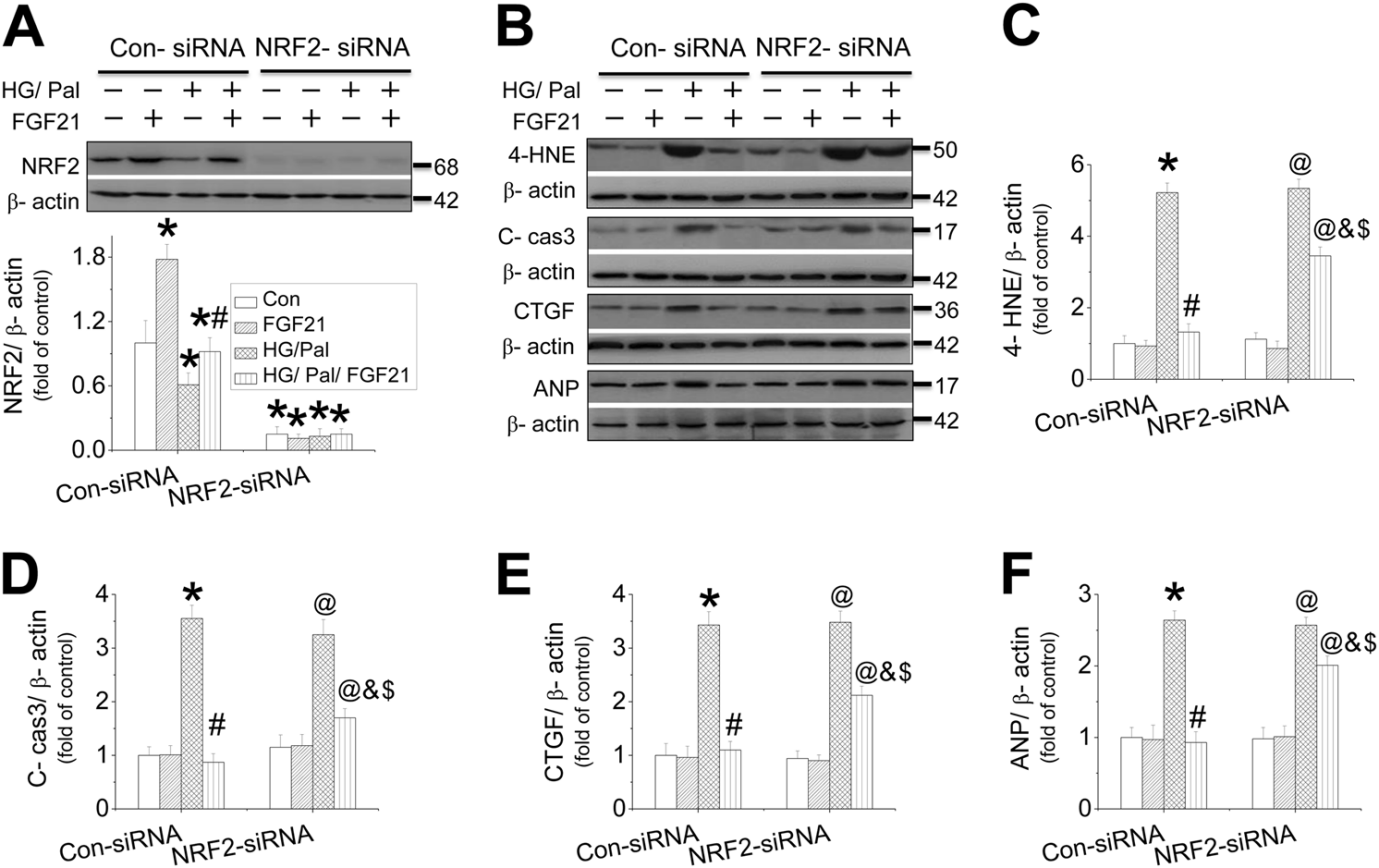
The role of NRF2 in FGF21-induced protection on cardiomyocytes against HG/Pal. Primary cardiomyocytes were isolated and treated with either control or NRF2-specific siRNA and then the cells were cotreated with both HG/Pal and FGF21 for 24 h. Western blotting was used to detect NRF2 expression (**a**). The expressions of 4-HNE (B&C), cleaved-caspase 3 (**b**, **d**), CTGF (**b**, **e**), and ANP (**b**, **f**) were measured by western blot assay. Data were collected from at least three independent experiments and presented as mean ± SD. *P < 0.05 vs. control in the Con-siRNA group; ^#^*P* < 0.05 vs. HG/Pal in the Con-siRNA group; ^@^*P* < 0.05 vs. control in NRF2-siRNA group; ^&^*P* < 0.05 vs. HG/Pal in the NRF2-siRNA group; ^$^*P* < 0.05 vs. HG/Pal/ FGF21 in the Con-siRNA group

We found that FGF21 protected cardiomyocytes from HG/Pal associated with maintenance of NRF2’s antioxidative function. Thus, the role of NRF2 in FGF21-induced cardiac protection was investigated in mouse neonatal cardiomyocytes in the presence of NRF2-siRNA. NRF2-siRNA effectively reduced the expressions of NRF2 (Fig. 4a) and its downstream target genes expression in cardiomyocytes (Fig. S18A−C). Surprisingly, the knockdown of NRF2 just partially blocked FGF21-induced inhibition on oxidative stress (Fig. 4b, c and S18D), apoptosis (Fig. 4b, d), fibrosis (Fig. 4b, e and S18E), and cell hypertrophy (Fig. 4b, f and S18F) in neonatal mouse cardiomyocytes in the presence of HG/Pal. Consistent results were confirmed in adult mouse cardiomyocytes showing that knockdown of NRF2 (Fig. S19A) partially blocked FGF21-induced inhibition on cardiomyocyte apoptosis (Fig. S19B), fibrosis (Fig. S19C), and cell hypertrophy (Fig. S19D) in the presence of HG/Pal.

### AKT pathway mediates FGF21-induced cardiomyocyte protection via enhancement of NRF2’s function

**Fig. 5:**
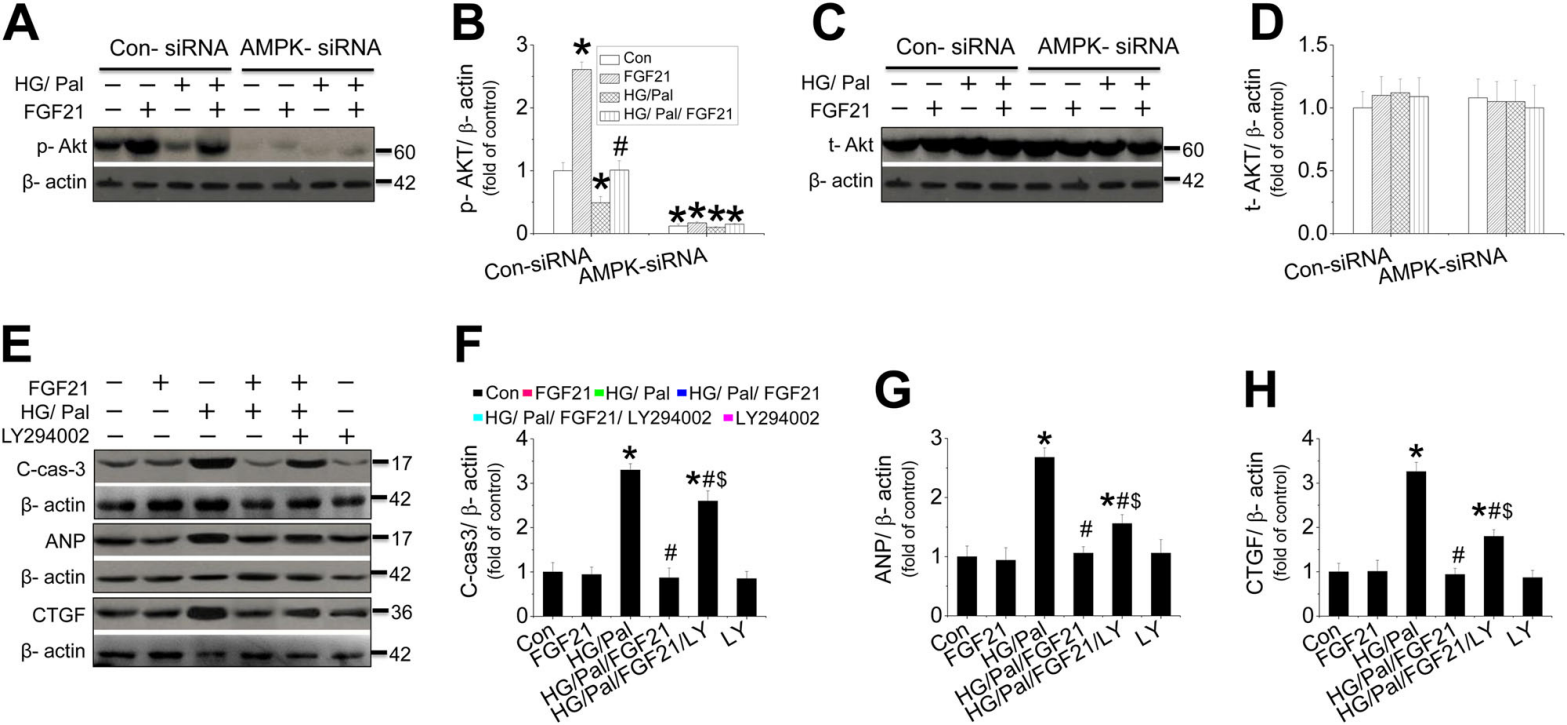
The impacts of exogenous FGF21 and AMPK on cardiac AKT-signaling pathway in the diabetic heart. AKT phosphorylation (**a**, **b**) and expression (**c**, **d**) in the primary cardiomyocytes were measured with or without AMPK-siRNA in the presence of HG/Pal. PI3K/AKT inhibitor (LY294002) was used to identify the role of AKT signaling in FGF21-induced cardiomyocyte protection by detecting the expression of cleaved-caspase-3 (**e**, **f**), ANP (**e**, **g**), and CTGF (**e**, **h**) by western blot. Data were collected from at least three independent experiments and presented as mean ± SD. For (**a**−**d**): *P < 0.05 vs. control in the Con-siRNA group. For (**e**−**h**) *P < 0.05 vs. control; ^#^*P* < 0.05 vs. HG/Pal; ^$^*P* < 0.05 vs. HG/Pal/FGF21

Knockdown of AMPK efficiently inhibited AKT phosphorylation (Fig. 5a, b) rather than AKT expressions (Fig. 5c, d) in mouse neonatal cardiomyocytes. Additionally, compared with WT mice, FGF21 deficiency further decreased the phosphorylation of cardiac AKT (Fig. S20A) and GSK-3β (Fig. S20B) induced by T2DM, associated with the upregulation of TRB3 expression (Fig. S20C) and PTEN activity (Fig. S20D) which are the negative regulators of AKT. However, FGF21 supplement rescued AKT signaling (Fig. S20A, B) by suppressing its negative regulators (Fig. S20C, D) in diabetic hearts. Interestingly, cardiomyocytes lost FGF21’s protection against apoptosis (Fig. 5e, f, Fig. S21A, B), hypertrophy (Fig. 5e, g, Fig. S21A, C), and fibrosis (Fig. 5e, h, Fig. S21A, D) in the presence of PI3K inhibitor (LY294002) or direct AKT inhibitor (10-DEBC hydrochloride), indicating that AKT signaling is required for FGF21-induced cardiac protection. The study in adult mouse cardiomyocytes also confirmed that FGF21-induced cardiomyocyte protection was partially inhibited by AKT inhibitor (Fig. S22).

**Fig. 6:**
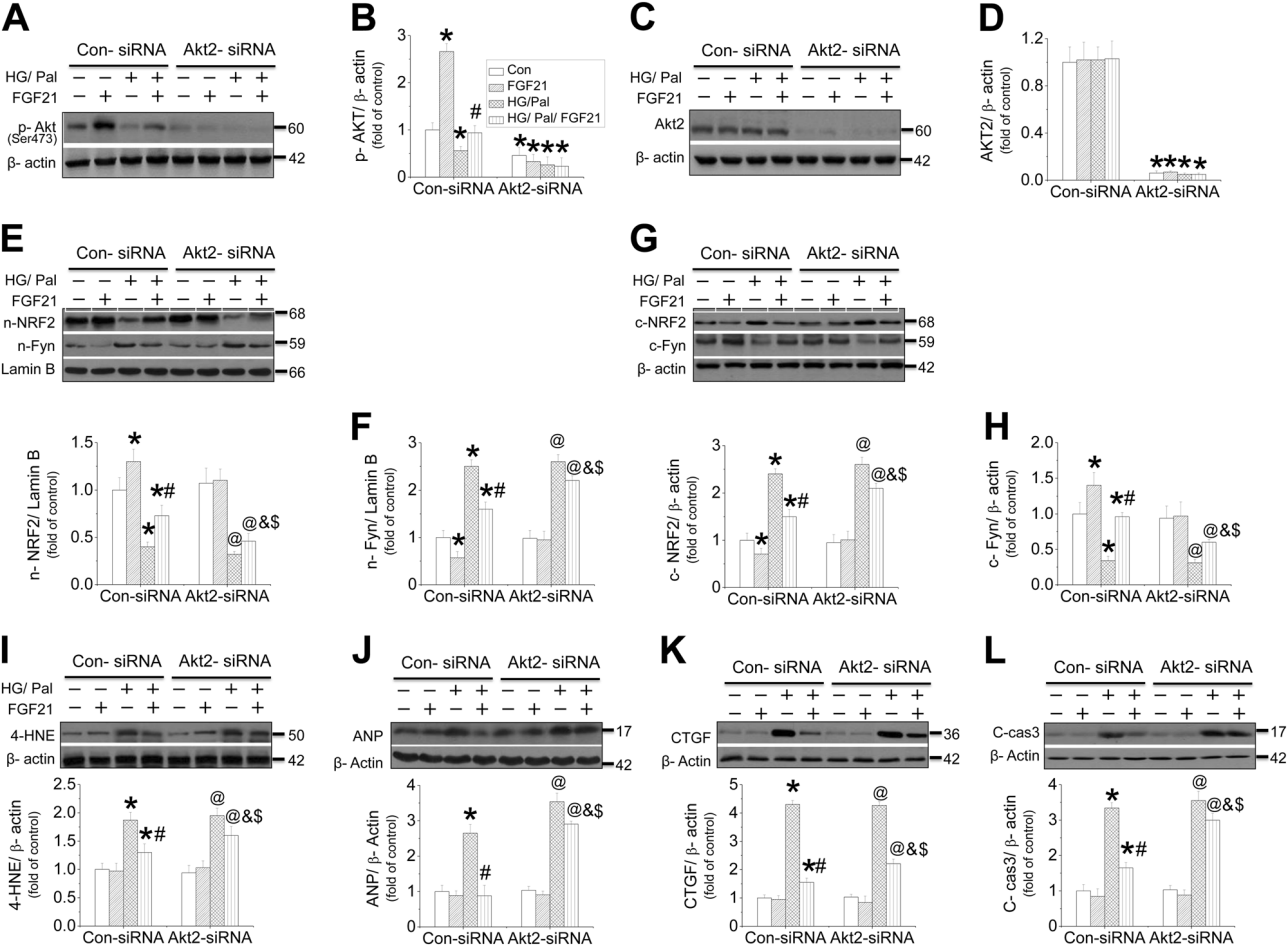
The role of AKT2 in FGF21-induced protection on cardiomyocytes against HG/Pal. Primary cardiomyocytes were isolated and treated with either control or AKT2-siRNA and then the cells were cotreated with both HG/Pal and FGF21 for 24 h. Western blotting was used to detect AKT phosphorylation (**a**, **b**) and AKT2 expression (**c**, **d**). The content of nuclear NRF2, Fyn (**e**, **f**), and cytosol NRF2, Fyn (**g**, **h**), and the expressions of 4-HNE (**i**), ANP (**j**), CTGF (**k**), and cleaved-caspase 3 (**l**) were also measured by western blot. Data were collected from at least three independent experiments and presented as mean ± SD. *P < 0.05 vs. control in the Con-siRNA group; ^#^*P* < 0.05 vs. HG/Pal in the Con-siRNA group; ^@^*P* < 0.05 vs. control in AKT2-siRNA group; ^&^*P* < 0.05 vs. HG/Pal in the AKT2-siRNA group; ^$^*P* < 0.05 vs. HG/Pal/FGF21 in the Con-siRNA group

Furthermore, siRNA for either AKT1, 2, or 3 was used to identify which subtype of AKT undertakes FGF21’s cardiomyocyte protection against HG/Pal. The results showed that AKT2-siRNA efficiently inhibited both AKT phosphorylation (Fig. 6a, b) and AKT2 expression (Fig. 6c, d). HG/Pal treatment strongly and comparably enhanced Fyn nuclear accumulation, but inhibited NRF2 nuclear translocation (Fig. 6e−h) and its downstream gene expressions (Fig. S23A−D), associated with induction of oxidative stress (Fig. 6i and S23E), cell hypertrophy (Fig. 6j and S23F, G), fibrosis (Fig. 6k and S23H), and apoptosis (Fig. 6l) in both Con-siRNA and AKT2-siRNA-treated cardiomyocytes. The cardiomyocyte protection of FGF21 against the above injuries was partially suppressed by AKT2-siRNA associated with reduced NRF2 nuclear translocation (Fig. 6 and S23). A similar finding was also observed in adult mouse cardiomyocytes that inhibition of AKT phosphorylation and expression using AKT2-siRNA (Fig. S24A−C) partially blocked FGF21-induced cardiomyocyte protection (Fig. S24D−H) and NRF2 nuclear translocation (Fig. S24G, I). However, knockdown of AKT1 or AKT3 using their siRNAs significantly blocked AKT1 (Fig.S25A, B) or AKT3 expression (Fig.S25D, E), respectively, but had no impact on FGF21-induced NRF2 nuclear translocation (Fig. S25E, F), suggesting that FGF21-induced cardiomyocyte protection against HG/Pal was partially mediated by AKT2–NRF2 pathway.

### AMPK-mediated lipid-lowering effect in the hearts is involved in FGF21-induced cardiac protection

**Fig. 7:**
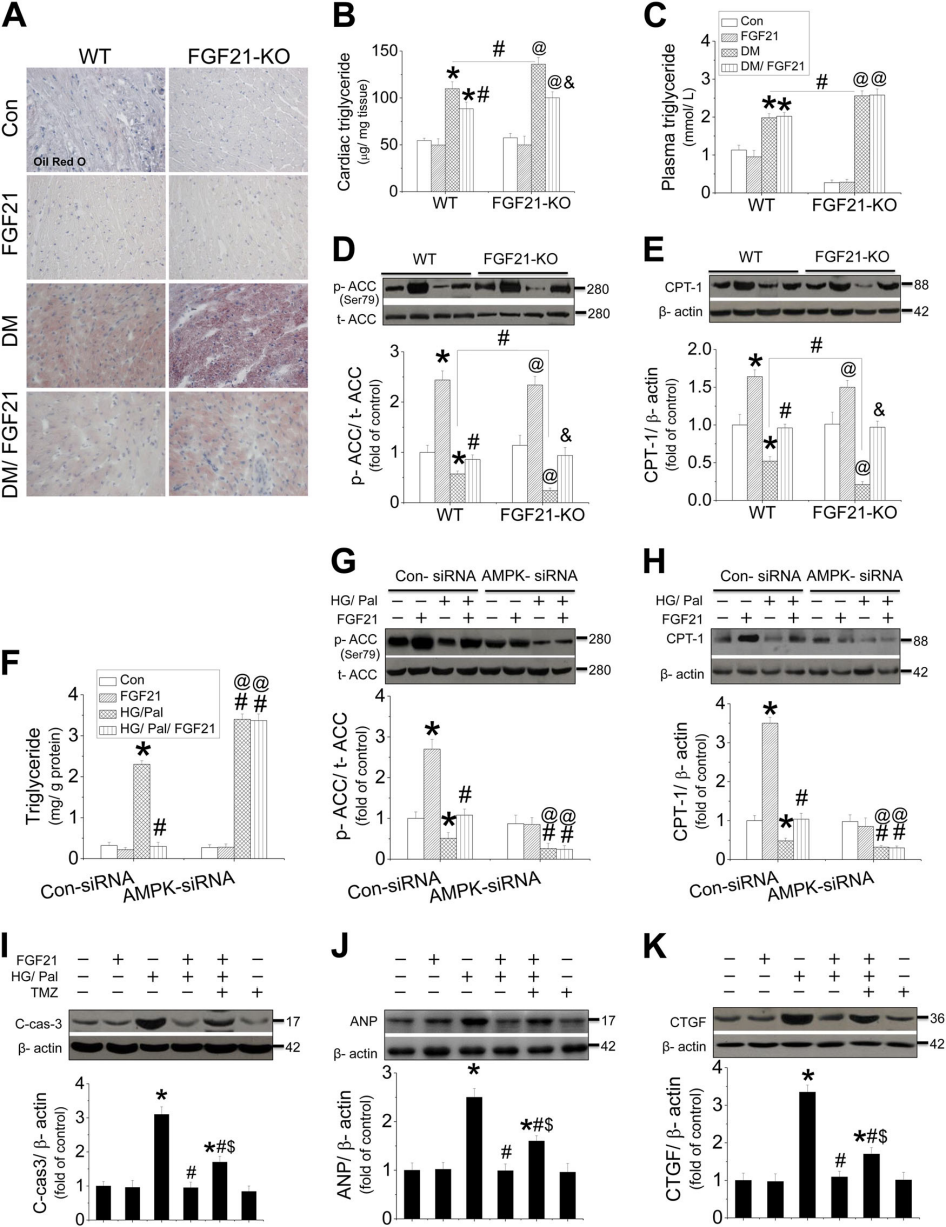
The effect and mechanism of FGF21 on diabetes-induced lipid accumulation in the heart. The slices of the heart from both WT and FGF21-KO mice were used for oil-O-red staining to evaluate lipid accumulation in the myocardium (**a**). Meanwhile, triglyceride levels of both the heart and plasma were examined by ELISA (**b**, **c**). Cardiac tissue was used for measuring ACC phosphorylation (**d**) and CPT-1 expression (**e**) by western blot to evaluate the activity of fatty acid β-oxidation pathway. Primary cardiomyocytes from AMPK-knockdown study were collected for detecting triglyceride by ELISA assay (**f**), as well as ACC phosphorylation (**g**) and CPT-1 expression (**h**) by western blot assay. Fatty acid β-oxidation inhibitor (TMZ) was used to identify the role of AMPK-mediated β-oxidation in FGF21-induced cardiomyocyte protection by detecting cleaved-caspase-3 level (**i**), ANP (**j**), and CTGF expressions (**k**) by western blot. For in vivo study (**a**−**e**), data are presented as means ± SD, *n* = 8/group. *P < 0.05 vs. the Con group in WT mice; ^#^*P* < 0.05 vs. the DM group in WT mice; ^@^*P* < 0.05 vs. the Con group in FGF21-KO mice; ^&^*P* < 0.05 vs. the DM group in FGF21-KO mice. For in vitro study (**f**−**h**), data were collected from at least three independent experiments and presented as mean ± SD. *P < 0.05 vs. control in the Con-siRNA group; ^#^*P* < 0.05 vs. HG/Pal in the Con-siRNA group; ^@^*P* < 0.05 vs. control in the AMPK-siRNA group; ^#^*P* < 0.05 vs. HG/Pal in the AMPK-siRNA group. For in vitro study (**i**−**k**), data were collected from at least three independent experiments and presented as mean ± SD. *P < 0.05 vs. control; ^#^*P* < 0.05 vs. HG/Pal; ^$^*P* < 0.05 vs. HG/Pal/FGF21

In the present study, we found that AMPK mediated FGF21’s protection on cardiomyocytes against HG/Pal partially via activation of AKT-NRF2 pathway; therefore, other protective mechanisms of FGF21 mediated by AMPK must exist. Oil red O staining showed that FGF21 deficiency further enhanced cardiac lipid accumulation and the increase of cardiac triglyceride level and plasma triglyceride level (Fig. 7a−c). In contrast, FGF21 supplement suppressed cardiac lipid accumulation and cardiac triglyceride level (Fig. 7a, b), but had no impact on plasma triglyceride level (Fig. 7c), suggesting that FGF21-induced lipid-lowering effect in the diabetic heart is independent of plasma triglyceride control. However, FGF21 reduces cardiac lipid accumulation against HG/Pal associated with improvement of ACC–CPT-1-mediated fatty acid β-oxidation pathway (Fig. 7d, e). FGF21-induced lipid-lowering effect was also confirmed in HG/Pal-treated neonatal mouse cardiomyocytes, characterized by decreased cellular triglyceride (Fig. 7f), associated with restoration of ACC phosphorylation (Fig. 7g) and CPT-1 expression (Fig. 7h). But the above phenomenon was completely blocked by AMPK-siRNA (Fig. 7f−h). A similar study was also performed in adult mouse cardiomyocytes that showed that FGF21-upregulated ACC–CPT-1 pathway was also mediated by AMPK (Fig. S26). Furthermore, inhibition of fatty acid β-oxidation by trimethazidine (TMZ) in the cardiomyocytes partially blocked FGF21-induced protection against apoptosis (Fig. 7i), hypertrophy (Fig. 7j), and fibrosis (Fig. 7k), suggesting that AMPK–ACC–CPT-1-mediated lipid metabolic pathway might contribute to FGF21 inducing lipid-lowering effect in the diabetic hearts.

As we know, cellular lipid accumulation is determined by the balance of lipid metabolism and lipid cellular absorption. Therefore, besides lipid metabolism, we also investigated the impact of FGF21 on lipid absorption of cardiomyocytes in the presence of HG/Pal. FGF21 deficiency further enhanced the expressions of CD36 and FATP, mediators of lipid absorption, in cardiomyocytes, which were strongly inhibited by FGF21 supplement (Fig. S27).

### Erk1/2-p 38MAPK pathway is required in FGF21-induced activation of AMPK

We previously demonstrated that FGF21 induced antiapoptotic effect in the hearts at the early stage of T1DM via activation of Erk1/2-p38 MAPK–AMPK signaling pathway. Therefore, in the present study, we investigated whether this pathway is also required in FGF21-induced prevention on cardiomyopathy in T2DM mice. The results showed that FGF21 increased, but diabetes decreased the phosphorylation of Erk1/2 (Fig. S28A, B) and p38 MAPK (Fig. S28A, C). Knockdown of Erk1/2 with Erk1/2-siRNA not only blocked Erk1/2 phosphorylation (Fig. S28D), but also significantly inhibited FGF21-induced upregulation of p38 MAPK and AMPK phosphorylation in the presence of HG/Pal (Fig. S28E, F). Moreover, knockdown of p38 MAPK with its siRNA inhibited the phosphorylation of both p38 MAPK and AMPK (Fig. S28G−H), indicating that Erk1/2-p38 MAPK–AMPK pathway is also involved in FGF21-induced cardiac protection against T2DM.

## Discussion

Clinical and preclinical studies have demonstrated that patients with cardiovascular diseases (CVDs) are always closely associated with serum FGF21 increase^27–29^. Furthermore, growing evidence indicated that FGF21 supplement induces preventive effects on these above CVDs^10,30–33^. As we know, the cardiovascular system is also the attacking target of diabetes. Our previous study proved that both endogenous and exogenous FGF21 prevent cardiac apoptosis in T1DM mice by inhibition of lipotoxicity^12^. Compared to T1DM, lipotoxicity is more severe in T2DM that is predominant in the clinic. Therefore, identifying the role of FGF21 in T2DM-induced DCM is more important and meaningful. However, it is unreasonable to predict the effect of FGF21 in T2DM hearts just based on the beneficial effect of FGF21 in T1DM mice, since etiological differences between the two types complicate the pathogenesis of the respective associated cardiomyopathies^26,34^. For example, compared to T2DM, systolic dysfunction is less to be observed in T1DM^26,34^. The different insulin actions (insulin resistance in T2DM vs. insulin deficiency in T1DM) may explain phenotypic differences in DCM^26,34^. In addition, a clinical study indicated that plasma FGF21 level decreases in T1DM patients but elevates in T2DM patients, implying that the action way of FGF21 might be different in the two types of diabetes^20^. Based on the above evidence and consideration, in the present study, the effect of FGF21 on DCM was performed in HFD/STZ-induced T2DM mice. The results indicated that FGF21 deficiency enhanced T2DM-induced cardiomyopathy characterized by cardiac dysfunction, remodeling, and myocardial morphological abnormalities. But all the above symptoms were remarkably prevented by FGF21 supplement, indicating that both endogenous and exogenous FGF21 induces cardiac protection against T2DM.

Interestingly, we found that FGF21-induced protection in the T2DM hearts was specifically associated with enhanced clearance of cardiac lipid rather than plasma lipid. And similar cardiac protection was found in DIO mice as observed in HFD/STZ-induced T2DM mice. Furthermore, an in vitro study showed that FGF21 supplement protected the cardiomyocytes against lipotoxicity rather than glucose toxicity, indicating that FGF21-induced cardiac protection in T2DM mice is mainly attributed to the prevention of lipotoxicity rather than hyperglycemia.

Next, we focused on dissecting the protective mechanism of FGF21 against T2DM-induced cardiomyopathy. Our previous study proved that Erk1/2-p38 MAPK–AMPK pathway mediates FGF21-induced antiapoptotic effect in the T1DM heart^12^. And this pathway also participates in FGF21-induced prevention on T2DM cardiomyopathy observed in the present study, indicating that inhibition of cardiac apoptosis contributes to the prevention of FGF21 on DCM. How does AMPK mediate FGF21’s cardiac protection in T2DM mice? As we know, diabetes induces mitochondrial dysfunction that causes the generation of excessive ROS^35,36^. ROS is the inducer of oxidative stress, which is the initial pathogenesis of cardiac apoptosis and the subsequent DCM^37,38^.

An in vitro study demonstrated that FGF21 protected the cardiac cell against hydrogen peroxide via suppression of oxidative stress^39^. Additionally, in both the liver and the brain, FGF21 prevented d-galactose-induced oxidative stress via activation of NRF2 pathway^40,41^. Moreover, Planavila et al. revealed that FGF21 protected the heart from oxidative stress induced by lipopolysaccharide or isoproterenol via induction of multiple antioxidative genes transcription^10^. In the present study, both exogenous and endogenous FGF21 induce antioxidative effect in T2DM hearts attributed to activation of NRF2 that regulates cellular resistance of oxidative stress by induction of an array of antioxidant genes expression^19,42^.

How does FGF21 regulate cardiac NRF2’s activity? Strong evidence demonstrated that activation of AKT-GSK3β prevents Fyn-mediated export of NRF2 from the nucleus that restores NRF2 nuclear-binding ability^43^. Our previous study also showed that fenofibrate attenuated diabetic renal damage through activation of FGF21-stimulated Akt/GSK-3β/Fyn pathway and enhanced NRF2-mediated antioxidative function^19^. In an in vitro study, knockdown of AKT2, but not AKT1 or AKT3, inhibited NRF2 nuclear translocation and function induced by FGF21 in cardiomyocytes. Furthermore, AKT2-siRNA partially suppressed FGF21-induced beneficial effects against cell hypertrophy, fibrosis, and apoptosis induced by HG/Pal, implying that AKT2–NRF2-induced antioxidative pathway partially mediated the cardiac protection of FGF21 against DCM.

**Fig. 8:**
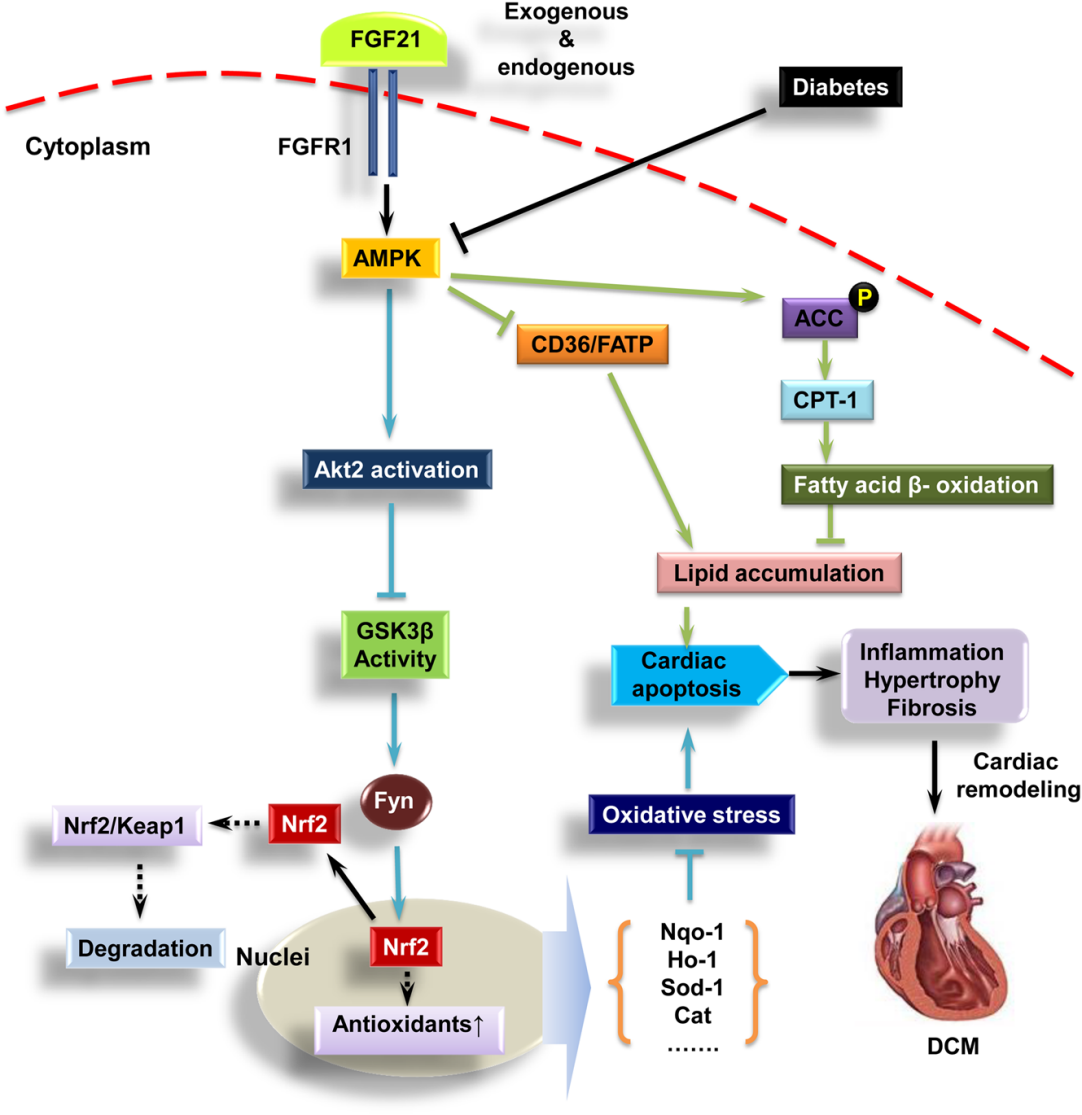
The protective mechanism of FGF21 on type 2 diabetes-induced cardiomyopathy. Heart is a special organ that predominantly consumes fatty acid to generate energy. Compared to glucose, more oxygen is required for generating an equal amount of adenosine triphosphate. Under diabetic conditions, glucose metabolism is impaired that forces the heart to consume more fatty acid to generate energy. During this period, excessive ROS is produced and accumulates in the cardiac cells that induce oxidative stress. Oxidative stress will cause cardiac cell injury and apoptosis followed by cardiac remodeling and finally leads to cardiac dysfunction and heart failure. The present study showed that both exogenous and endogenous FGF21 induces the preventive effect on type 2 diabetes-induced cardiomyopathy. Further study reveals that the cardiac protection of FGF21 was mediated by AMPK. First, AMPK activates AKT2–GSK3β pathway that enhances NRF2 nuclear translocation via inhibition of nuclear accumulation of Fyn to induce antioxidant genes expression. Second, AMPK also actives fatty acid β-oxidation via ACC–CPT-1 pathway to specifically reduce lipid accumulation in the myocardium. Therefore, both antioxidative and lipid-lowering pathways regulated by AMPK mediate FGF21’s prevention on DCM

So, the next question is how does FGF21 activate AKT signaling in T2DM heart? Strong evidence demonstrated that pyrrolidinyl caffeamide prevents ischemia/reperfusion injury in cardiomyocytes through AMPK/AKT pathways^22^. Activation of AMPK/AKT pathway suppressed the endothelial dysfunction induced by OxLDL^21^. Our previous studies showed that AMPK mediated FGF21’s cardiac protection against T1DM via inactivation of phosphatase and tensin homolog deleted on chromosome ten (PTEN) which is a negative regulator of AKT^12^. In the present study, knockdown of AMPK suppressed AKT-NRF2 pathway and it mediated cardiomyocyte protection induced by FGF21, suggesting that AMPK mediated FGF21-induced cardiac protection against DCM partially through activation of AKT2–NRF2-induced antioxidative pathway (Fig. 8).

Interestingly, AKT2–NRF2 antioxidant pathway just partially contributes to AMPK-mediated FGF21’s cardiac protection against T2DM, suggesting that other protective mechanisms of FGF21 mediated by AMPK must exist. Further study showed that lipid-lowering effect also contributes to FGF21-induced cardiomyocyte protection against HG/Pal via improvement of fatty acid β-oxidation. Knockdown of AMPK blocked FGF21-induced ACC–CPT-1-mediated fatty acid β-oxidation and the following lipid-lowering effect of FGF21 in cardiomyocytes, revealing that AMPK-mediated lipid-lowering effect is also involved in the preventive effect of FGF21 against DCM (Fig. 8).

As we know, the cellular lipid accumulation is determined by the balance of lipid clearance and lipid absorption. In the present study, we found that besides enhancement of lipid clearance (lipid metabolism), FGF21 supplement also suppressed the elevated expressions of CD36 and FATP, regulators for lipid cellular absorption, in the diabetic heart in both WT and FGF21-KO mice, indicating that FGF21 reduces cardiac lipid accumulation by both fatty acid oxidation enhancement and lipid absorption inhibition.

Mouse neonatal cardiomyocytes have the disadvantage to mimic adult murine cardiac response. Accordingly, we isolated the cardiomyocytes from adult mouse to perform the main experiments that reflect the cardiomyocyte protective effect and the protective mechanisms of FGF21. The results confirmed the conclusion of the neonatal cardiomyocyte studies, that FGF21 treatment prevented Pal/HG-induced cardiomyocyte damage including oxidative stress, hypertrophic change, inflammation, and apoptosis via activation of AMPK-mediated antioxidative pathway and lipid-lowering pathway in adult mouse cardiomyocytes. In summary, we confirmed that FGF21 induces preventive effect on T2DM-induced cardiomyopathy characterized by improving cardiac function, suppressing cardiac remodeling, and apoptosis. A mechanistic study indicated that FGF21-induced cardiac protection against DCM is probably mediated by AMPK-induced antioxidative pathway (AKT–GSK3β–Fyn–NRF2) and lipid-lowering pathway (ACC–CPT-1) (Fig. 8).

## Materials and methods

### Establishment of both T2DM mouse model and DIO mice, and FGF21 treatment

T2DM was established in both FGF21 knockout (FGF21-KO, gift from Dr. Steve Kliewer, University of Texas Southwestern Medical Center) and age-matched WT (C57BL/6J background, male, 8-weeks old) mice by using HFD plus streptozotocin (STZ) injection strategy as indicated previously^31^. An equal amount and treating time of HFD were applied in WT mice without STZ injection and will be induced in DIO mice. Then, the diabetic and DIO mice intraperitoneally received recombinant FGF21 (100 μg/kg/day, synthesized in our laboratory by gene engineering^44^) treatment for 4 months. See supplementary methods for details.

### Noninvasive blood pressure

Blood pressure (BP) including diastolic and systolic BPs was measured by tail-cuff manometry^5,6,15^. See supplementary methods for details.

### Measurement of cardiac function by echocardiography

Cardiac function was measured by echocardiography^12,45^. See supplementary methods for details.

### Biochemical parameters

Serum insulin levels were measured with insulin ELISA kit (Crystal Chem, Downers Grove, IL, USA). Plasma triglyceride and cholesterol concentrations were measured by using triglyceride assay kit (Cayman Chemicals, Ann Arbor, MI). Plasma FGF21 was determined with an FGF21 Quantikine Elisa kit (R&D Systems, Minneapolis, MN, USA).

### Glucose tolerance test and insulin tolerance test

To assess glucose tolerance, mice were intraperitoneally injected with d-glucose (1.5 g/kg) after an overnight fasting (12 h) with free access to water, and venous blood was collected 30 min before (time 0) and after injection at 0, 15, 30, 60, and 120 min from the tail of each mouse, and glucose was measured using a FreeStyle glucose meter. To assess insulin tolerance, a single dose of Novolin R regular insulin (Novo Nordisk A/S, Denmark) (0.5 units/kg or 1 unit/kg) was intraperitoneally administered to the mice after fasting for 4 h with free access to water, and the blood glucose level was measured as described above.

### Evaluation of CH

CH of mice was evaluated by three methods^42,46^: (1) measurement of LV mass; (2) calculating the relative heart weight; and (3) detecting the changes of CH markers. See supplementary methods for details.

### Morphological examination of the hearts

Myocardium slices from the mice in each group were stained with H&E for detection of morphological changes^31^, with Sirius-red for detection of collagen accumulation (fibrosis)^5^, or with oil-O-red for detection of lipid accumulation^47^, respectively. See supplementary methods for details.

### Terminal deoxynucleotidyl transferase-mediated dUTP nick end labeling (TUNEL) assay

The ApopTag Peroxidase in situ Apoptosis Detection Kit (Chemicon, Temecula, CA, USA) was applied for the TUNEL staining^48^. See supplementary methods for details.

### Detection of cardiac malondialdehyde production

A thiobarbituric acid assay was used to measure the relative malondialdehyde production as an index of lipid peroxidation^42^. See supplementary methods for details.

### Intracellular ROS measurement

The ROS production was measured by using the ROS-sensitive dye, 2,7-dichlorodihydrofluorescein diacetate (DCF-DA, Invitrogen) as an indicator^49,50^. See supplementary methods for details.

### RNA isolation and real-time quantitative polymerase chain reaction (PCR)

RNA isolation and RT-PCR were performed as described in our previous studies^31,51^. See supplementary methods for details.

### Nuclei isolation

Nuclei of the cardiomyocytes from both in vivo and in vitro studies were isolated using nuclei isolation kit (NUC-201, Sigma, MO, USA) as previously described^47^. See supplementary methods for details.

### Isolation and treatment of cardiomyocytes

Neonatal/adult mouse cardiomyocytes were isolated as described^52–54^. The isolated cardiomyocytes were transfected with either negative control sense siRNA or target siRNA using Lipofectamine TM 2000 (Invitrogen, Carlsbad, CA) transfection reagent for 48 h as described by the manufacturer^42^. Then, the cardiomyocytes were exposed to d-glucose (27.5 mm was added to reach the final concentration of 33 mm; high glucose, HG) for 24 h along with FGF21 treatment (50 ng/ml). Palmitate (Pal, 62.5 μmol/l) was added during the last 15 h (totally 24 h for HG/Pal treatment). See supplementary methods for details.

### Western blotting assay

Western blot was performed as described in our previous studies^31,51^. See supplementary methods for details.

### Statistical analysis

Data were collected from eight mice per group, or three replicates of cell-culture experiments, which presented as mean ± standard deviation (SD). One-way ANOVA was used to determine general differences, followed by a post hoc Tukey’s test for the difference between groups, using Origin 7.5 software for laboratory data analysis and graphing. Statistical significance was considered *P* < 0.05.

## Electronic supplementary material

Supplementation-clean
