# Supplementary material for: Fibroblast growth factor-21 prevents diabetic cardiomyopathy via AMPK-mediated antioxidation and lipid-lowering effects in the heart
Source: Cell Death Dis. 2018 Feb 14;9(2):227. doi: 10.1038/s41419-018-0307-5 (PMC5833682; doi:10.1038/s41419-018-0307-5)
Supplement: Supplementary file 1 — Supplementation-clean [file 41419_2018_307_MOESM1_ESM.docx]

**Supplementary Materials and Methods**

***Ethics Statement***

The protocol was approved by the [Committee on the Ethics of Animal Experiments](http://www.google.com/url?q=http://en.wikipedia.org/wiki/Institutional_Animal_Care_and_Use_Committee&sa=U&ei=amUAUKerGerK6wGTqJ3DBw&ved=0CB8QFjAC&usg=AFQjCNF0kw6I2dKGG2PnmDUTi8fxrs-n1g) of the Wenzhou Medical University, Zhejiang, China. All surgery was performed under anesthesia induced by intraperitoneal injection of 1.2% 2,2,2-Tribromoethanol (Avertin) (Sigma-Aldrich, St. Louis, MO) at the dose of 0.2 ml/10 g body weight and all efforts were made to minimize suffering of the experimental animals.

***Establishment of type 2 diabetic mouse model and FGF21 treatment***

FGF21 knock-out (FGF21-KO) mice, 8 weeks old (18-22g of body weight) with a C57BL/6J genetic background (gift from Dr. Steve Kliewer, University of Texas Southwestern Medical Center). Age-matched WT C57BL/6J mice as controls were obtained from Jackson Laboratory. All mice were housed at 22°C with a 12:12-h light-dark cycle with free access to rodent chow and tap water. Animals were kept under these conditions for 2 weeks before the experiments.

In the present study, high-fat-diet (HFD) plus streptozotocin (STZ) injection strategy was applied to induce type 2 diabetes as described previously in both WT and FGF21-KO mice [^1^](#_ENREF_1). Firstly, mice were fed with HFD (Shanghai SLAC laboratory Animal Co., Ltd., 40% of calories from fat) for 12 weeks to induce obesity accompanied by insulin resistance. Then the obese mice were intraperitoneally given a single injection of streptozotocin (STZ) at 100 mg/kg body weight to induce hyperglycemia and establish type 2 diabetes. The age-matched non-diabetic mice were given an injection of equivalent volume of citrate buffer and fed with standard diet (SD, Shanghai SLAC laboratory Animal Co., Ltd., 10% of calories from fat). The DIO mice model was just fed with HFD without STZ injection. Diabetic and non-diabetic mice or DIO and non-DIO mice were intraperitoneally received recombinant FGF21 (100μg/ kg/ day, synthetized in our laboratory by gene engineering [^2^](#_ENREF_2)) treatment for 4 months.

***Non-invasive blood pressure***

Blood pressure (BP) was measured by tail-cuff manometry using CODATM non-invasive BP monitoring system (Kent Scientiﬁc Corporation, Torrington, CT). Mice were kept in warm on the heating pad to ensure its normal blood circulation after restrained in a plastic tube restrainer. Occlusion and volume-pressure recording cuffs were placed over the tail. Mice were allowed to adapt to the restrainer for 3 times prior to BP measurement. The BP was measured for 10 acclimation cycles followed by 20 measurement cycles. After three days of training for the BP measurement, formal measurements for the unanesthetized BP were collected [^3^](#_ENREF_3).

***Measurement of cardiac function by Echocardiography***

Transthoracic echocardiography (Echo) using a high-resolution imaging system for small animals (GE healthcare, Piscataway, NJ, USA), equipped with a 12-MHz microprobe. The cardiac function of each mouse was performed under anesthetized condition after chest hair was removed. The aquasonic clear ultrasound gel (Parker Laboratories, Fairfield, NJ) was applied to the surface of the thorax to optimize cardiac chamber visibility. Parasternal long-axis and short-axis views were acquired. LV end-diastolic diameter (LVID;d), LV end-systolic diameter (LVID;s), interventricular septal thickness in end-diastole (IVS;d), interventricular septal thickness in end-systole (IVS;s), left ventricular posterior wall thickness in end-diastole (LVPW;d), left ventricular posterior wall thickness in end-systole (LVPW;s), Ejection fraction percent (EF%), fractional shortening percent (FS%), left ventricular (LV) mass and corrected LV mass were calculated using GE healthcare software. Data are average values of 10 cardiac cycles [^4^](#_ENREF_4)^,^ [^5^](#_ENREF_5).

***Evaluation of CH***

CH of mice was evaluated by three methods [^6^](#_ENREF_6)^,^ [^7^](#_ENREF_7): (1) detecting and calculating LV mass and corrected LV mass using Echo; (2) calculating the ratio of HW to TL; (3) detecting the expressions of the protein markers of CH in the heart tissue including atrial natriuretic peptides (ANP), brain natriuretic peptide (BNP) and β-myosin heavy chain (β-MHC) by western-blotting assay.

***Morphological examination of the hearts***

Mice hearts were isolated after sacrificed. The middle part of the heart tissues by crosscutting were fixed with 10% formalin for 48 hours at room temperature. After dehydration in ethanol, the tissue blocks were embedded in paraffin, then cut into blocks 5 μm thick and stained with hematoxylin and eosin (H&E) for general morphological examination. Additionally, the slices were stained with 0.1% Sirius-red F3BA and 0.25% Fast green FCF for the collagen accumulation which reflecting cardiac fibrotic changes, as described in our previous study. The collagen content was determined by quantitative analysis of Sirius-red positive area using Image Pro software (Media Cybernetics, Silver Spring, Maryland). Lipid accumulation in the heart tissue was measured by Oil Red O staining as described previously [31]. Cryosections from optimal cutting temperature medium (OCT)-embedded tissue samples of the hearts (8-mm thick) were fixed in 10% buffered formalin for 5 minutes at room temperature followed by staining with Oil Red O reagent for 1 hour, washed with 10% isopropanol, and then counterstained with hematoxylin (DAKO, Carpinteria, CA) for 30 seconds. A Nikon microscope (Nikon, Melville, NY) was used to capture the oil red O-stained tissue sections.

***Terminal deoxynucleotidyl transferase-mediated dUTP nick end labeling (TUNEL) assay***

The ApopTag Peroxidase *in situ* Apoptosis Detection Kit (Chemicon, Temecula, CA, USA) was applied for the TUNEL staining [^8^](#_ENREF_8). The slides were deparaffinized and rehydrated, then treated with proteinase K (20 mg/L) for 15 min. After that the slices were treated with 3% hydrogen peroxide for 5 min to inhibit the endogenous peroxidase, and then incubated for one hour with terminal deoxynucleotidyl transferase (TdT) and digoxigenin-11-dUTP. The TdT reaction was carried out in a dark and humidified chamber at 37 °C for 15 minitutes. During the process 2×SSC was applied. Counterstaining was performed using 4',6-diamidino-2-phenylindole (DAPI). For the negative control, TdT was omitted from the reaction mixture. Apoptotic cell death was quantitatively analyzed by counting the TUNEL-positive cells selected randomly from 10 fields at ×40.

***Detection of cardiac malondialdehyde (MDA) production***

Briefly, total proteins of cardiac tissue were collected by centrifugation at 12,000 × *g* at 4 °C for 15 min followed by concentration determination using Bradford assay. Then, 50 μl of sample was mixed with 20 μl of 8.1% SDS, 150 μl of 20% acetic acid, and 210 μl of 0.0571% TBA, and incubated at 90 °C for 70 minutes. Samples were centrifuged at 4,000 rpm for 15 minutes at 4 °C, harvested, transferred to 96-well plates, and optical density was read at 540 nm. Data are expressed as nmol/mg protein.

***Intracellular ROS measurement***

The ROS production was measured by using the ROS-sensitive dye, 2,7-dichlorodihydro-fluorescein diacetate (DCF-DA, Invitrogen), as an indicator [^9^](#_ENREF_9). Samples (50 μg proteins) of hearts tissue were incubated with 10 μl of DCF-DA (10 μmol/l) for 3 h at 37°C. The fluorescent product formed was quantified by spectrofluorometer at the 485/525 nm [^10^](#_ENREF_10).

***RNA isolation and real-time quantitative polymerase chain reaction (PCR)***

Total RNA was isolated from hearts tissue using TRIzol reagent (Invitrogen, Carlsbad, CA). The concentration of total mRNA in each sample was quantified with a Nanodrop 2000 (Thermo Scientific, San Jose, CA). The mRNA samples were reverse transcribed into cDNA using a High-Capacity cDNA Reverse Transcription Kit (PE Applied Biosystems, Foster City, CA). The following primers were used for RT-PCR. *HO-1*: forward, 5’-CTCCCTGTGTTTCCTTTCTC-3’; and reverse, 5’-CTGCTGGTTTCAAAGTTCAG-3’; *NQO-1*: forward, 5’-GAGAAGAG -CCCTGATTGTAC-3’; and reverse, 5’-ACCTCCCATCCTCTCTTCTT-3’; *CAT*: forward 5′-TGAGAAGCCTAAGAACGCAA-3’ and reverse 5′-CCCTTCGCAGCC -ATGTG-3′; *SOD-1*: forward 5′-TTAACGCGCAGATCATGCA-3′ and reverse 5′-G -GTGGCGTTGAGATTGTTCA-3′;  *β-actin*: forward, 5’- AGGTATCCTGACCCT -GAAGT-3’; and reverse, 5’-CACACGCAGCTCATTGTAGA -3’. RT-PCR was carried out in triplicate using the SYBR GREEN PCR master mix (Invitrogen, Carlsbad, CA) on a Stratagene MX3000p thermocycler (Agilent StrataGene, Santa Clara, CA). The amount of mRNA was calculated by the comparative CT method, which depends on the ratio of the amount of target genes to reference gene *β-actin*.

***Nuclei isolation***

Nuclei of the cardiomyocytes from both *in vivo* and *in vitro* studies were isolated using nuclei isolation kit (NUC- 201, Sigma, MO, USA) as previously [^11^](#_ENREF_11). Briefly, 50 mg caridac tissues or 1 x 10^7^ cardiomyocytes were homogenized for 50 seconds. within 300 ml cold lysis buffer containing 1 ml dithiothreitol (DTT) and 0.1% Triton X-100. After that, 600 ml cold 1.8 mol/L Cushion Solution was add to the lysis solution. The mixture was transferred to a new tube pre-loaded with 300 ml 1.8 mol/L Sucrose Cushion Solution followed by a centrifugation at 30,000 × g for 45 minutes. The supernatant containing cytoplasmic component was saved for later analysis. Nuclei were visible as thin pellet at the bottom of tube.

***Isolation and treatment of cardiomyocytes***

Neonatal mouse cardiomyocytes were prepared as previously [^12^](#_ENREF_12). Briefly, hearts from 2-day-old mice were minced and dissociated with 0.15% trypsin. Dispersed cells were plated for 2 hours with minimum essential medium plus 10% bovine calf serum to remove noncardiomyocytes. The remained myocytes in the suspension were plated with the above medium plus 0.1 mmol/l bromodeoxyurindine, and 20 μmol/l arabinosylcytosine at a density of 500 cells/mm^2^. The medium was replaced by fresh medium after culturing for 24 hours. On day 3, the cardiomyocytes were incubated with 30 μg/ml lipopolysaccharide plus 100 ng/ml TNF-α for 48 hours, then the cardiomyocytes were ready to be used.

Adult mouse cardiomyocytes were isolated as described [^13^](#_ENREF_13)^,^ [^14^](#_ENREF_14)^,^ [^15^](#_ENREF_15). Mice (2 months-of-age) were anesthetized with ketamine (43.5 mg/kg), acepromazine (1.5 mg/kg), and xylazine (1.7 mg/kg), and heparin (100 U/ml). After median sternotomy,  the heart was quickly isolated and arrested in ice-cold Ca^2+^-Tyrode solution. Then hearts were perfused in the Langendorff mode on a gravity flow system at 37°C. Firstly hearts were perfused with a Ca^2+^-Tyrode solution aerated with 100% O_2_ (in mM: 137 NaCl, 5.4 KCl, 1.8 CaCl_2_, 0.5 MgCl_2_, 10 HEPES, 10 glucose, pH 7.4) to free the residual blood. After 5 min hearts were perfused with Ca^2+^-free Tyrode solution (in mM: 135 NaCl, 4 KCl, 1 MgCl_2_, 10 HEPES, 0.33 NaH_2_PO_4_, 10 BDM, 10 glucose, pH 7.2) for 3 minutes. Subsequently, hearts were digested by collagenase II (0.6 mg/ml) and pancreatin (0.08 mg/ml) dissolved in Ca^2+^-free Tyrode until a sudden increase in flow rate occurred, indicating effective tissue digestion. Then the heart tissue was removed, shredded, and filtered through a 140 µm nylon mesh. The supernatant was transferred to another tube filled with Krebs-Henseleit buffer (in mM: 0.5 EDTA, 5.1 KCl, 0.6 MgSO4, 118 NaCl, 1.2 KH2PO4, 10 glucose, 1 NaHCO3, 10 HEPES, 2 mg/ml BSA Fraction V, Sigma).and CaCl_2_ was added incrementally at 4-minute intervals (five total steps) to increase Ca^2+^ concentration to 500 µmol/l. The suspension was then plated on laminin-coated culture (Dulbecco’s modified Eagles medium with glucose at the concentration of 5.5 mmol/l, 10% FBS, 1% penicillin and 1% kanamycin)for 5 hours at 37 °C in a 5% CO_2_ incubator. Media was replaced and cultured for another 5 hours before experimentation to wash away unattached cells and ensure that only rod-shaped myocytes were used for subsequent studies (totally 10 h before formal experiment). The cardiomyocytes were transfected with either negative control sense siRNA or target siRNA using Lipofectamine TM 2,000 (Invitrogen, Carlsbad, CA) transfection reagent for 48 hours as described by the manufacturer. Then the cardiomyocytes were exposed to D-glucose (27.5 mM was added to reach the ﬁnal concentration 33 mM; high glucose, HG) for 24 hours associated with FGF21 treatment (50 ng/ ml). Palmitate (Pal, 62.5 μmol/L) was added during the last 15 hours (totally 24 hours for HG/ Pal treatment). Since as a saturated fatty acid, palmitate can not be directly absorbed by cells. Bovine serum albumin (BSA) acts as a carrier which can conjugated with palmitate and carries it into the cell for metabolism. Therefore, cell culture media with 2% bovine serum albumin (BSA, Sigma-Aldrich) was used during this period.

***Inhibitor treatment***

PI3K/ AKT inhibitor (LY294002) purchased form Sigma (St. Louis, MO) were dissolved in pure DMSO to 50μmol/ L. 10-DEBC, AKT direct inhibitor purchased form Tocris Bioscience (Ellisville, MO) were dissolved in pure DMSO to 20μmol/ L. [Trimethazidine](http://www.baidu.com/link?url=Nko-kyNZXPt4j82Q-lQRfr8t25V34lo4TlKylVIO_g7zTlWehrpb7Hswv3QjXt4uiPjASeE19eReJ_rRgV4kpEmptUCg4ck5bvLfmEpfCg0vf2hBBLCo_aLb-vIVC8N2CgasszoyZpvDXFej70aQZa) (TMZ), inhibitor for fatty acid β- oxidation, purchased form Sigma (St. Louis, MO) were dissolved in pure DMSO to 10μmol/ L. Isolated cardiomyocytes were pre-treated by LY294002, 10-DEBC or TMZ for 3 hours followed by co-treatment with HG/ Pal in the presence of FGF21 supplement for another 24 hours.

***siRNA transfection***

The siRNA transfections in the cardiomyocytes of both neonatal and adult mouse were performed using Lipofectamine^TM^ 2000 (Invitrogen, Carlsbad, CA) as previously [^5^](#_ENREF_5). Specifically, in order to knockdown AMPK, NRF2, AKT1/2/3, Erk1/2 and P38 MAPK in cardiomyocytes, cardiomyocytes were transfected with mouse non-targeting Stealth RNAi™ siRNA (120 nmol/l) of the above kinases along with the corresponding non-specific control siRNA (120 nmol/l) (Invitrogen, Carlsbad, CA). After 24h transfection of siRNA, cardiomycytes were pre-treated with HG (33mmol/l) and FGF21 (50 ng/ml) for another 24 hours. Palmitate (Pal, 62.5 μmol/L) was added during the last 15 hours (totally 24 hours for HG/ Pal treatment). The efficiency of siRNAs transfection was assessed by Western-blot analysis the expression and activity (phosphorylation) for AMPK, NRF2, AKT2, Erk1/2, and P38 MAPK.

***Western blotting assay***

Cardiac tissues were homogenized in lysis buffer (Santa Cruz Biotechnology, Santa Cruz, CA) and the supernatants were collected by centrifugation at 12,000 × g and 4 °C. Equal amounts of protein from each sample were separated on 10% SDS-PAGE and transferred to nitrocellulose membranes. After blocking with non-fat milk for 1 h at room temperature, membranes were incubated overnight at 4 °C with the following primary antibodies: atrial natriuretic peptide (ANP, 1:1,000), brain natriuretic peptide (BNP, 1:1,000), β-myosin heavy chain (β-MHC, 1:1,000), connective tissue growth factor (CTGF, 1:2,000), 3-Nitrotyrosine (3-NT, 1:2,000), 4-Hydroxynonenal (4-HNE, 1:1,000), Nuclear factor (erythroid-derived 2)-like 2 (NRF2, 1:1,000) and β-actin (1:1,000) purchased from Abcam (Cambridge, MA). Phosphorylated-P53 (p-P53, 1:1,000), total-P53 (t-P53, 1:1,000), cleaved-caspase-3 (C-cas3, 1:500), phosphorylated-protein kinase B (p-AKT, 1:1,000), AKT1, 2 or 3 (1:1,000), phosphorylated-glycogen synthase kinase-3β (GSK-3β, 1:1,000), total-GSK-3β (1:1,000), Fyn (1:1,000), tumor necrosis factor factor α (TNFα, 1:1,000), nuclear factor-kappa B p65 subunit (NF-κB p65, 1:1,000), inhibitor of NF-κB (I-κB, 1:1,000) , phosphatase and tensin homolog deleted on chromosome ten (PTEN, 1:1000), Tribbles 3 (TRB3), Acetyl-CoA carboxylase (ACC), [carnitine palmitoyl transterase-1(CPT-1)](http://www.baidu.com/link?url=DYAogIs5ea_BiJD62PsXBAInYDbEgL71IKnEJaeTFpJVzF_oUtoNkZe1YcoLqQzovtdbmK5qgNAnknmI9W8P2a), CD36 (1:1000), fatty acid trans portprotein (FATP, 1:1000), extracellular regulated protein kinases (Erk)1/2 (1:2000) and p38 MAPK (1:1000) purchased from Cell Signaling Technology (Danvers, MA). Intercellular adhesion factor-1 (ICAM-1, 1:1000) and β- actin (1:1000) wpurchased form Santa Cruz Biotechnology (Dallas, TA, USA). Plasminogen activator inhibitor-1 (PAI-1) purchased form BD Biosciences (San Jose, CA, USA). After three washes in Tris-buffered saline containing 0.05% Tween 20 (TBST), the membranes were incubated with horseradish peroxidase-conjugated secondary antibodies for 1 h at room temperature. Antigen-antibody complexes were then visualized using an enhanced chemiluminescence kit (Amersham, Piscataway, NJ), and the intensity of the protein bands was quantified using Quantity one software (Version 4.6.2, Bio-Rad).

***Statistical analysis***

Data was collected from 8 mice per group, or 3 replicates of cell-culture experiments, which presented as mean ± standard deviation (SD). One-way ANOVA was used to determine general differences, followed by a post-hoc Tukey’s test for the difference between groups, using Origin 7.5 software for laboratory data analysis and graphing. Statistical significance was considered *P* < 0.05.

**References**

1. Shao M, Lu X, Cong W, Xing X, Tan Y, Li Y*, et al.* Multiple low-dose radiation prevents type 2 diabetes-induced renal damage through attenuation of dyslipidemia and insulin resistance and subsequent renal inflammation and oxidative stress. *PloS one* 2014, **9**(3)**:** e92574.

2. Wang H, Xiao Y, Fu L, Zhao H, Zhang Y, Wan X*, et al.* High-level expression and purification of soluble recombinant FGF21 protein by SUMO fusion in Escherichia coli. *BMC biotechnology* 2010, **10:** 14.

3. Bai Y, Cui W, Xin Y, Miao X, Barati MT, Zhang C*, et al.* Prevention by sulforaphane of diabetic cardiomyopathy is associated with up-regulation of Nrf2 expression and transcription activation. *Journal of molecular and cellular cardiology* 2013, **57:** 82-95.

4. Basu R, Oudit GY, Wang X, Zhang L, Ussher JR, Lopaschuk GD*, et al.* Type 1 diabetic cardiomyopathy in the Akita (Ins2WT/C96Y) mouse model is characterized by lipotoxicity and diastolic dysfunction with preserved systolic function. *American journal of physiology Heart and circulatory physiology* 2009, **297**(6)**:** H2096-2108.

5. Zhang C, Huang Z, Gu J, Yan X, Lu X, Zhou S*, et al.* Fibroblast growth factor 21 protects the heart from apoptosis in a diabetic mouse model via extracellular signal-regulated kinase 1/2-dependent signalling pathway. *Diabetologia* 2015, **58**(8)**:** 1937-1948.

6. Zhang F, Lin X, Yu L, Cheng P, He L, Yang H*, et al.* Low-dose radiation prevents type 1 diabetes-induced cardiomyopathy via activation of AKT mediated anti-apoptotic and anti-oxidant effects. *Journal of cellular and molecular medicine* 2016.

7. Zhang C, Wang F, Zhang Y, Kang Y, Wang H, Si M*, et al.* Celecoxib prevents pressure overload-induced cardiac hypertrophy and dysfunction by inhibiting inflammation, apoptosis and oxidative stress. *Journal of cellular and molecular medicine* 2016, **20**(1)**:** 116-127.

8. Cai L, Wang Y, Zhou G, Chen T, Song Y, Li X*, et al.* Attenuation by metallothionein of early cardiac cell death via suppression of mitochondrial oxidative stress results in a prevention of diabetic cardiomyopathy. *Journal of the American College of Cardiology* 2006, **48**(8)**:** 1688-1697.

9. Shen E, Li Y, Li Y, Shan L, Zhu H, Feng Q*, et al.* Rac1 is required for cardiomyocyte apoptosis during hyperglycemia. *Diabetes* 2009, **58**(10)**:** 2386-2395.

10. Tanajak P, Sa-Nguanmoo P, Wang X, Liang G, Li X, Jiang C*, et al.* Fibroblast growth factor 21 (FGF21) therapy attenuates left ventricular dysfunction and metabolic disturbance by improving FGF21 sensitivity, cardiac mitochondrial redox homoeostasis and structural changes in pre-diabetic rats. *Acta physiologica* 2016, **217**(4)**:** 287-299.

11. Zhang C, Lu X, Tan Y, Li B, Miao X, Jin L*, et al.* Diabetes-induced hepatic pathogenic damage, inflammation, oxidative stress, and insulin resistance was exacerbated in zinc deficient mouse model. *PloS one* 2012, **7**(12)**:** e49257.

12. Cai L, Wang J, Li Y, Sun X, Wang L, Zhou Z*, et al.* Inhibition of superoxide generation and associated nitrosative damage is involved in metallothionein prevention of diabetic cardiomyopathy. *Diabetes* 2005, **54**(6)**:** 1829-1837.

13. Luo J, Hill BG, Gu Y, Cai J, Srivastava S, Bhatnagar A*, et al.* Mechanisms of acrolein-induced myocardial dysfunction: implications for environmental and endogenous aldehyde exposure. *American journal of physiology Heart and circulatory physiology* 2007, **293**(6)**:** H3673-3684.

14. Sambrano GR, Fraser I, Han H, Ni Y, O'Connell T, Yan Z*, et al.* Navigating the signalling network in mouse cardiac myocytes. *Nature* 2002, **420**(6916)**:** 712-714.

15. Pinz I, Zhu M, Mende U, Ingwall JS. An improved isolation procedure for adult mouse cardiomyocytes. *Cell biochemistry and biophysics* 2011, **61**(1)**:** 93-101.

**Supplemental table 1. Effect of FGF21 on cardiac function in mice with diabetic cardiomyopathy**

|  | **Con** | **FGF21** | **DM** | **DM/ FGF21** |
| --- | --- | --- | --- | --- |
| **Diastolic BP**  **(mm Hg)** | 76.54 ± 1.66 | 73.76 ± 2.62 | 79.64 ± 3.52 | 76.31 ± 1.15 |
| **Systolic BP**  **(mm Hg)** | 107.65 ± 2.75 | 104.68 ± 1.49 | 110.44 ± 3.53 | 108.65 ± 3.21 |
| **LVID;d (mm)** | 3.64 ± 0.21 | 3.61 ± 0.32 | 4.08 ± 0.27* | 3.71 ± 0.15*^#^ |
| **LVID;s (mm)** | 1.65 ± 0.15 | 1.62 ± 0.21 | 2.25 ± 0.15* | 1.91 ± 0.17^#^ |
| **IVS;d (mm)** | 0.71 ± 0.06 | 0.73 ± 0.05 | 0.84 ± 0.05* | 0.72 ± 0.02^#^ |
| **IVS;s (mm)** | 1.15 ± 0.06 | 1.14 ± 0.08 | 0.81 ± 0.04* | 1.12 ± 0.06^#^ |
| **LVPW;d (mm)** | 0.85 ± 0.03 | 0.82 ± 0.02 | 1.26 ± 0.09* | 0.90 ± 0.05*^#^ |
| **LVPW;s (mm)** | 1.82 ± 0.14 | 1.81 ± 0.11 | 1.34 ± 0.12* | 1.64 ± 0.11*^#^ |
| **%EF (%)** | 86.21 ± 1.66 | 88.23 ± 1.12 | 61.49 ± 1.52* | 76.82 ± 1.04*^#^ |
| **%FS (%)** | 61.33 ± 1.16 | 61.63 ± 1.01 | 41.42 ± 1.17* | 55.02 ± 1.03*^#^ |
| **LV mass (mg)** | 91.54 ± 2.46 | 90.43 ± 1.58 | 111.57 ± 1.44* | 100.64 ± 2.42*^#^ |
| **LV mass-C (mg)** | 75.15 ± 1.77 | 75.36 ± 1.26 | 89.53± 2.54* | 83.41 ± 1.22*^#^ |

Notes: Data are presented as means ± SD, n = 8/ group. **P* < 0.05 *vs*. the control (Con) group; ^#^*P* < 0.05 *vs.* diabetic (DM) group.

**Supplemental table 2. Effect of FGF21 deficiency on blood pressure and cardiac function in type 2 diabetic mice**

|  | **Con (WT)** | **Con (KO)** | **DM (WT)** | **DM (KO)** | **DM (KO)/ FGF21** |
| --- | --- | --- | --- | --- | --- |
| **Diastolic BP**  **(mm Hg)** | 74.33± 2.21 | 76.86± 3.13 | 82.43 ± 3.28 | 84.59 ± 2.52 | 81.55 ± 2.17 |
| **Systolic BP**  **(mm Hg)** | 106.55± 2.17 | 107.47± 2.64 | 114.37 ± 3.29 | 115.35 ± 2.25 | 113.15 ± 3.63 |
| **LVID;d (mm)** | 3.54 ± 0.27 | 3.59 ± 0.11 | 4.18 ± 0.58* | 6.12 ± 0.67^@$^ | 4.01 ± 0.23^@&^ |
| **LVID;s (mm)** | 1.62 ± 0.11 | 1.68 ± 0.22 | 2.35 ± 0.19* | 3.65 ± 0.22^@$^ | 2.05 ± 0.13^@&^ |
| **IVS;d (mm)** | 0.57 ± 0.02 | 0.52 ± 0.08 | 0.82 ± 0.09* | 1.05 ± 0.05^@$^ | 0.72 ± 0.06^@&^ |
| **IVS;s (mm)** | 1.21 ± 0.05 | 1.17± 0.07 | 0.83 ± 0.03* | 0.65 ± 0.06^@$^ | 0.93 ± 0.06^@&^ |
| **LVPW;d (mm)** | 0.86 ± 0.07 | 0.85 ± 0.05 | 1.27 ± 0.04* | 1.54 ± 0.09^@$^ | 1.16 ± 0.05^@&^ |
| **LVPW;s (mm)** | 1.82 ± 0.19 | 1.87 ± 0.14 | 1.38 ± 0.16* | 1.01 ± 0.08^@$^ | 1.51 ± 0.12^@&^ |
| **%EF (%)** | 87.11 ± 1.45 | 84.16 ± 1.42 | 61.39 ± 1.22* | 54.11 ± 1.05^@$^ | 66.12 ± 1.36^@&^ |
| **%FS (%)** | 63.31 ± 1.28 | 61.18 ± 1.31 | 42.12 ± 1.15* | 36.17 ± 1.22^@$^ | 48.33 ± 1.33^@&^ |
| **LV mass (mg)** | 92.14 ± 1.36 | 91.34 ± 2.41 | 110.37 ± 1.94* | 119.54 ± 1.44^@$^ | 102.32 ± 1.93^@&^ |
| **LV mass-C (mg)** | 73.12 ± 1.71 | 74.15 ± 1.63 | 88.93± 2.11* | 96.93± 2.92^@$^ | 80.13± 2.56^@&^ |

Notes: Data are presented as means ± SD, n = 8/ group. **P* < 0.05 vs. the Con (WT) group; ^@^*P* < 0.05 vs. the Con (KO) group; ^&^*P* < 0.05 vs. the DM (KO) group; ^$^*p* < 0.05 vs. the DM (WT) group

**Supplemental table 3. Effect of FGF21 on cardiac function in mice with obesity**

|  | **Con** | **FGF21** | **DIO** | **DIO/ FGF21** |
| --- | --- | --- | --- | --- |
| **Diastolic BP**  **(mm Hg)** | 77.33 ± 1.54 | 74.12 ± 1.87 | 79.44 ± 1.29 | 78.12 ± 3.21 |
| **Systolic BP**  **(mm Hg)** | 104.63 ± 1.73 | 103.48 ± 1.39 | 106.17 ± 3.22 | 105.16 ± 1.47 |
| **LVID;d (mm)** | 3.54 ± 0.26 | 3.60 ± 0.21 | 4.19 ± 0.17* | 3.72 ± 0.12*^#^ |
| **LVID;s (mm)** | 1.62 ± 0.16 | 1.60 ± 0.11 | 2.24 ± 0.14* | 1.92 ± 0.14^#^ |
| **IVS;d (mm)** | 0.73 ± 0.05 | 0.72 ± 0.03 | 0.85 ± 0.01* | 0.71 ± 0.04^#^ |
| **IVS;s (mm)** | 1.17 ± 0.08 | 1.16 ± 0.08 | 0.84 ± 0.03* | 1.11 ± 0.05^#^ |
| **LVPW;d (mm)** | 0.79 ± 0.05 | 0.77 ± 0.03 | 1.23 ± 0.04* | 0.92 ± 0.02*^#^ |
| **LVPW;s (mm)** | 1.83 ± 0.13 | 1.83 ± 0.11 | 1.31 ± 0.15* | 1.60 ± 0.16*^#^ |
| **%EF (%)** | 86.13 ± 1.32 | 85.16 ± 1.04 | 62.55 ± 1.57* | 77.09 ± 1.18*^#^ |
| **%FS (%)** | 61.76 ± 1.18 | 61.61 ± 1.06 | 43.66 ± 1.25* | 56.32 ± 1.13*^#^ |
| **LV mass (mg)** | 92.55 ± 2.27 | 91.65 ± 1.82 | 110.25 ± 1.61* | 100.40 ± 2.11*^#^ |
| **LV mass-C (mg)** | 76.09 ± 1.53 | 75.17 ± 1.66 | 88.57 ± 2.53* | 82.21 ± 1.53*^#^ |

Notes: Data are presented as means ± SD, n = 8/ group. **P* < 0.05 *vs*. the control (Con) group; ^#^*P* < 0.05 *vs.* DIO group.

**
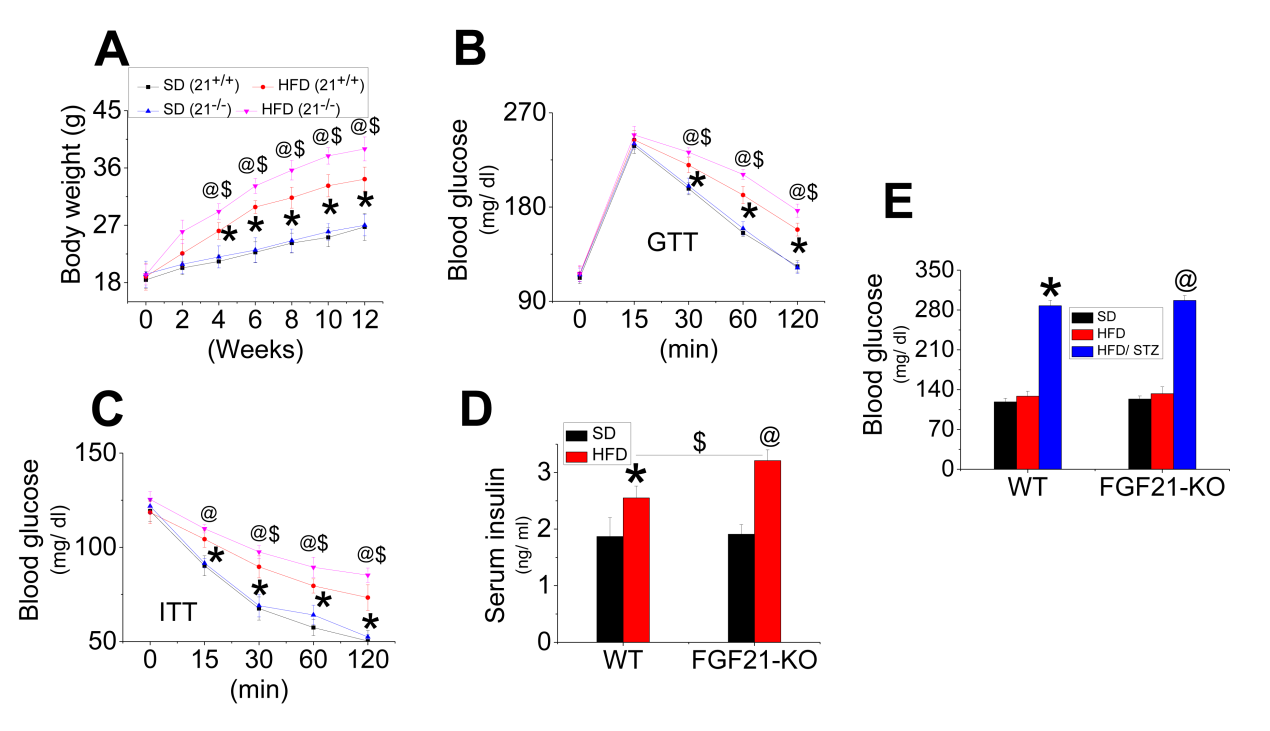
**

**Figure S1. Establishment of type 2 diabetic mouse model using the HFD/STZ.** WT and FGF21 mice were fed with HFD for 12 weeks to induce obesity. Body weight (A), glucose tolerance (B), and insulin sensitivity (C) and plasma insulin level were examined. Five days after STZ injection blood glucose was measured (D). The mice were regarded as diabetic once hyperglycemia was observed (> 250 mg/ dL). Data are presented as means ± SD; *n* = 8 per group. **P* < 0.05 *vs.* the SD group (WT); ^@^*P* < 0.05 *vs.* the SD group (KO); ^$^*P* < 0.05 *vs.* the HFD group (WT). HFD, high-fat diet; SD, standard food; diabetes mellitus; GTT, glucose tolerance test; ITT, insulin tolerance test.


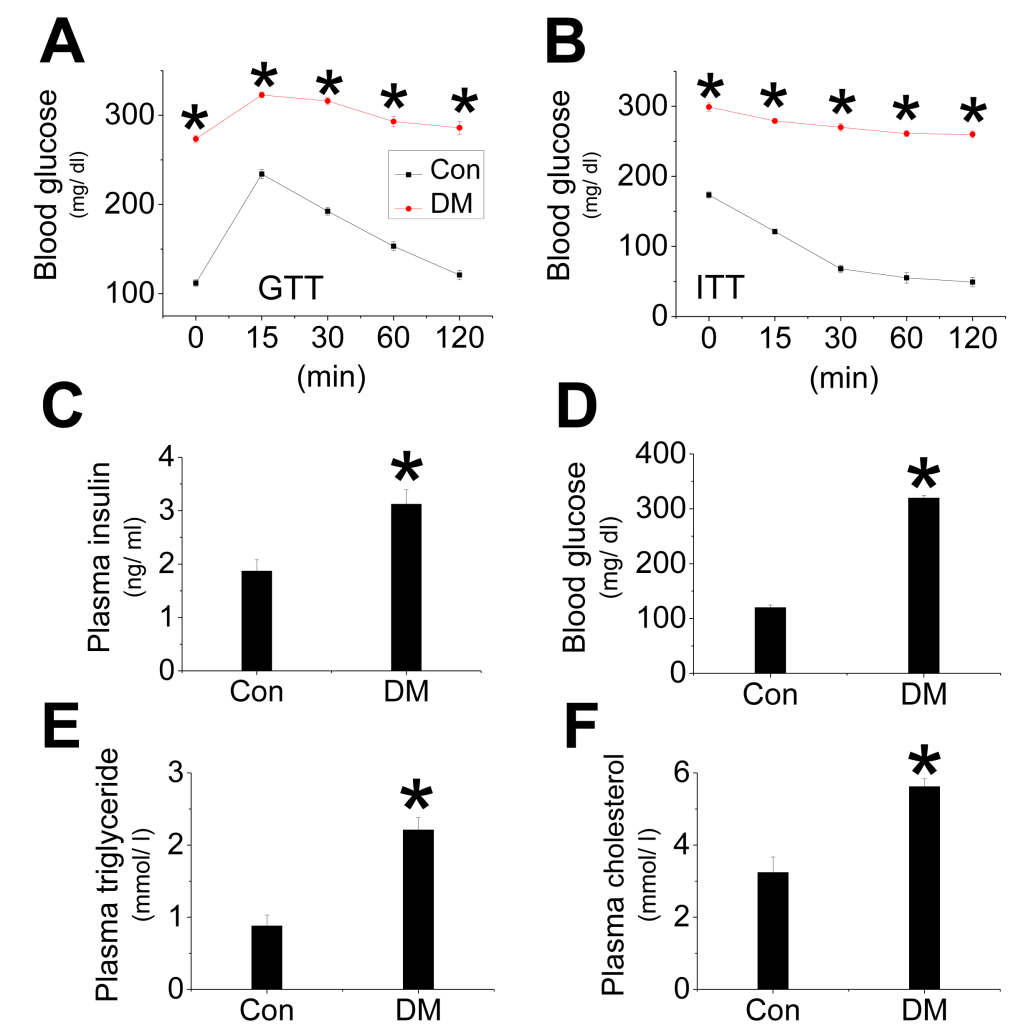


**Figure S2. The changes of the metabolic parametes in the mice with 4-month diabetes.** Four months after the T2DM established, the insulin resistance was determined by examination of the glucose tolerance (A), insulin sensitivity (B) and plasma insulin level (C) were examined. Moreover, the blood glucose level (D), plasma triglyceride (E) and cholesterol (F) were examined by ELISA. Data are presented as means ± SD; *n* = 8 per group. **P* < 0.05 *vs.* the non-diabetic group.

**
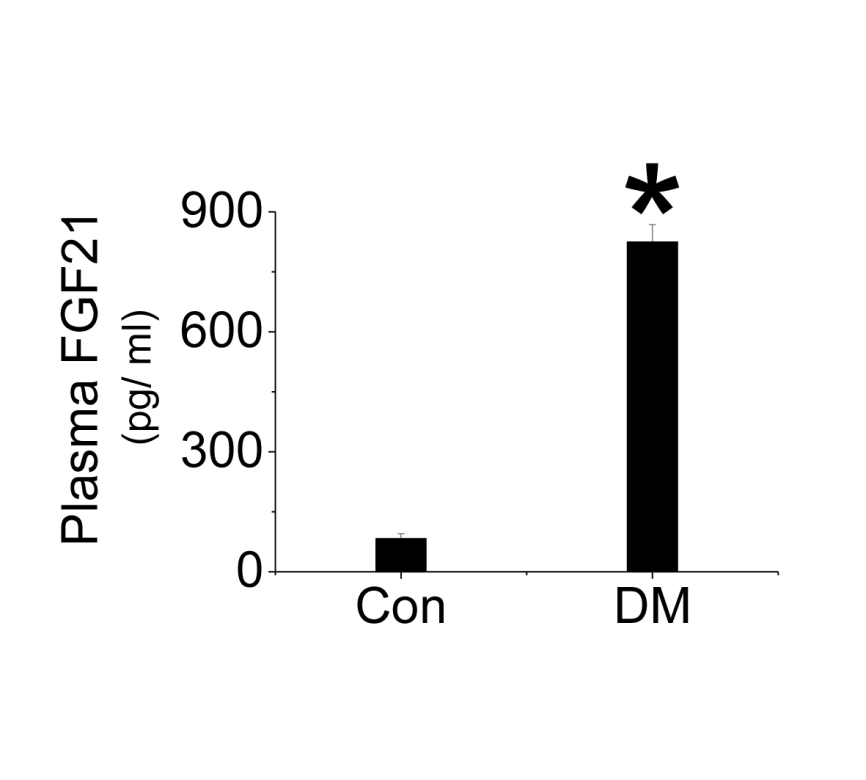
**

**Figure S3. Plasma FGF21 level was increased in diabetic mice.** Four months after the T2DM established, The plasma was collected and the plasma FGF21 of both non-diabetic and diabetic mice were determined by ELISA. Data are presented as means ± SD; *n* = 8 per group. **P* < 0.05 *vs.* the non-diabetic group.

**
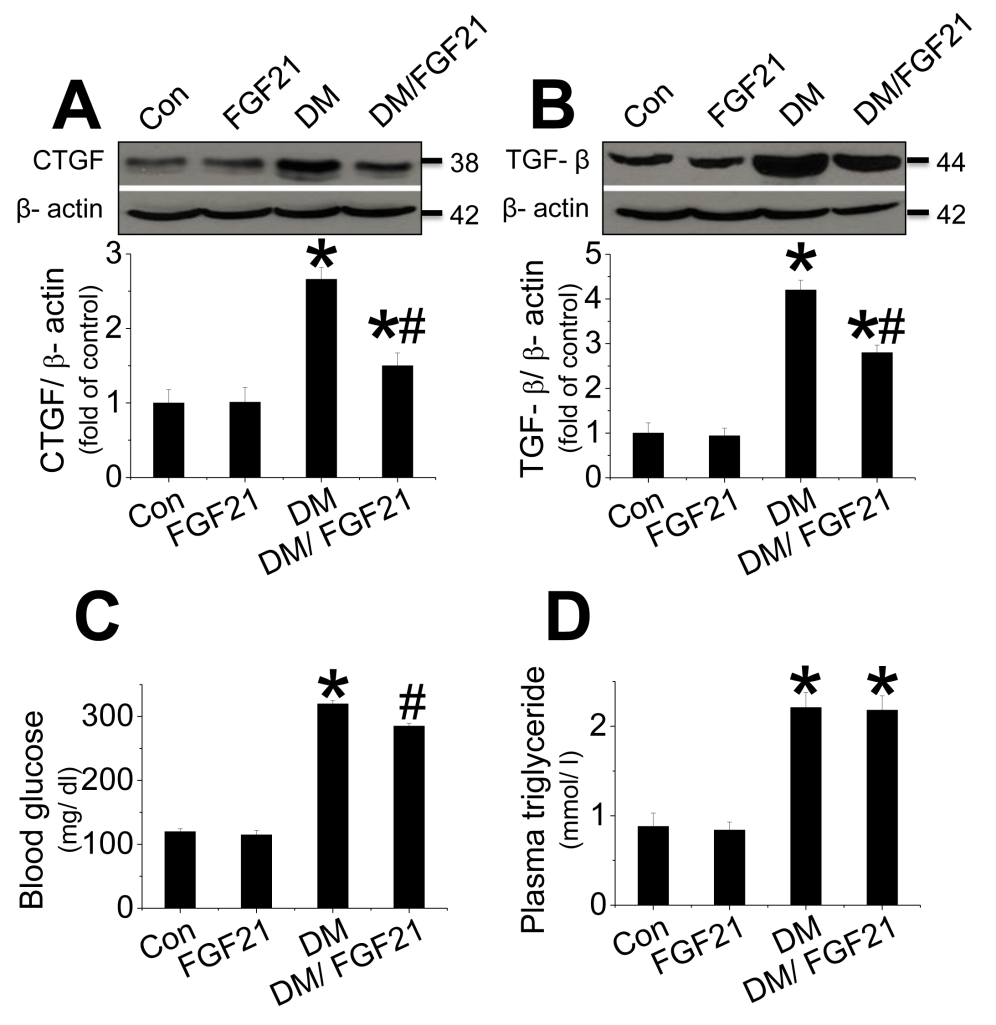
**

**Figure S4. Effect of FGF21 supplement cardiac fibrosis and blood glucose triglyceride level.** HFD/ STZ-induced type 2 diabetic and age-matched mice received FGF21 treatment for 4 months. Then mice were sacrificed and the hearts were isolated. The expression of fibrotic markers such as CTGF (A) and TGF-β (B) in diabetic hearts by Western-blot assay. Additionally, blood glucose level (C) and plasma triglyceride (D) were determined by ELISA. Data are presented as means ± SD, n = 8/ group. **P* < 0.05 *vs*. the control (Con) group; ^#^*P* < 0.05 *vs.* diabetic (DM) group.

**
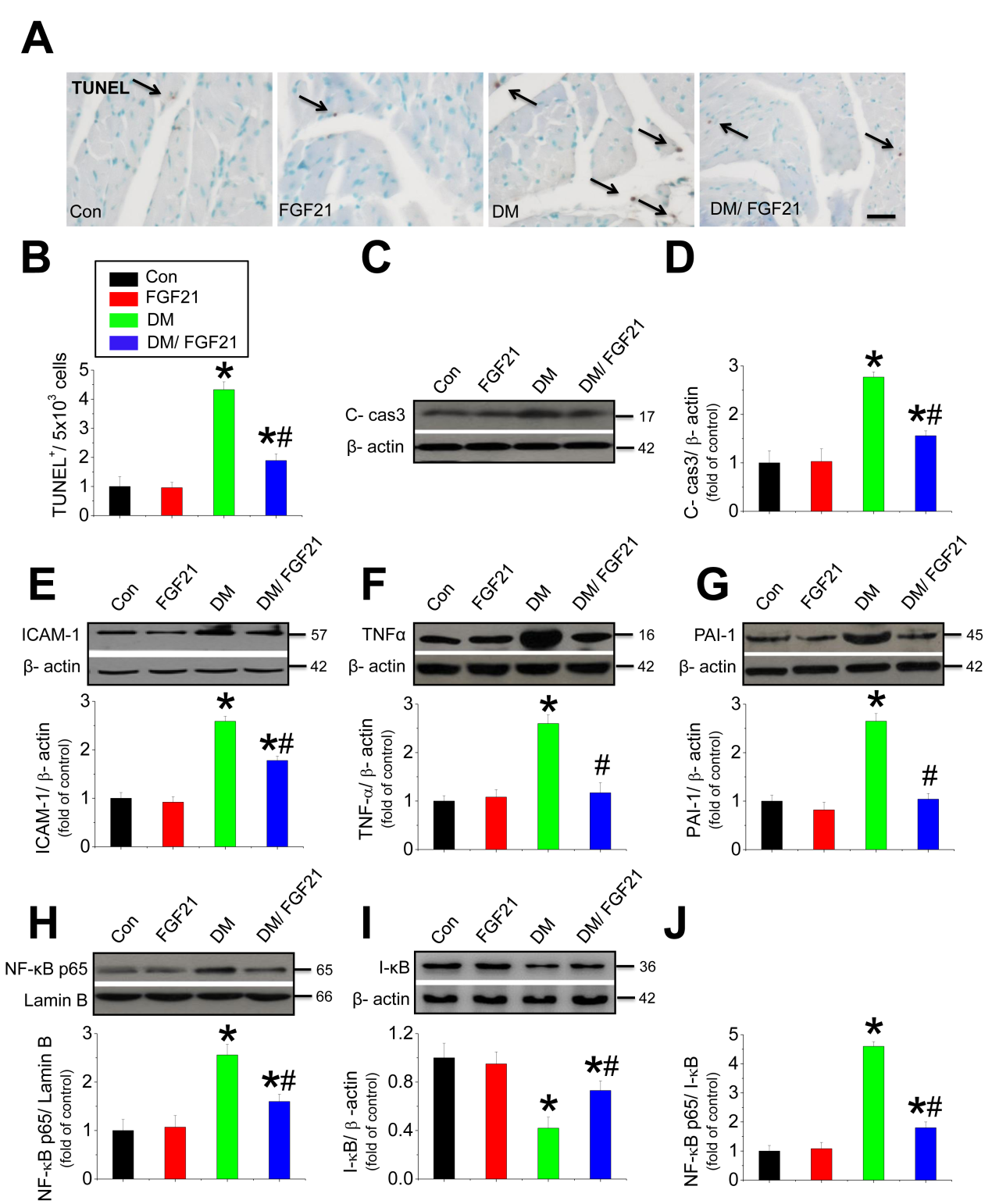
**

**Figure S5. Effect of FGF21 supplement on type 2 diabetes-induced cardiac apoptosis and inflammation.** Apoptosis was measured by TUNEL staining (A), followed by quantitative analysis of TUNEL-positive cells (B). Then cardiac apoptosis was further confirmed by measuring cleaved-caspase-3 expression (C&D) with Western blot. Additionally, same assay was applied to detect cardiac inflammation by measuring the expressions of multiple inflammatory factors including ICAM-1 (E), TNF-α (F) and PAI-1 (G). Expressions of NF-κB p65 (H), IκB (I), and their ratio (J) were examined by Western blot which are the key regulators of inflammation. Data are presented as means ± SD, n = 8/ group. **P* < 0.05 *vs*. the Con group; ^#^*P* < 0.05 *vs.* the DM group.

**
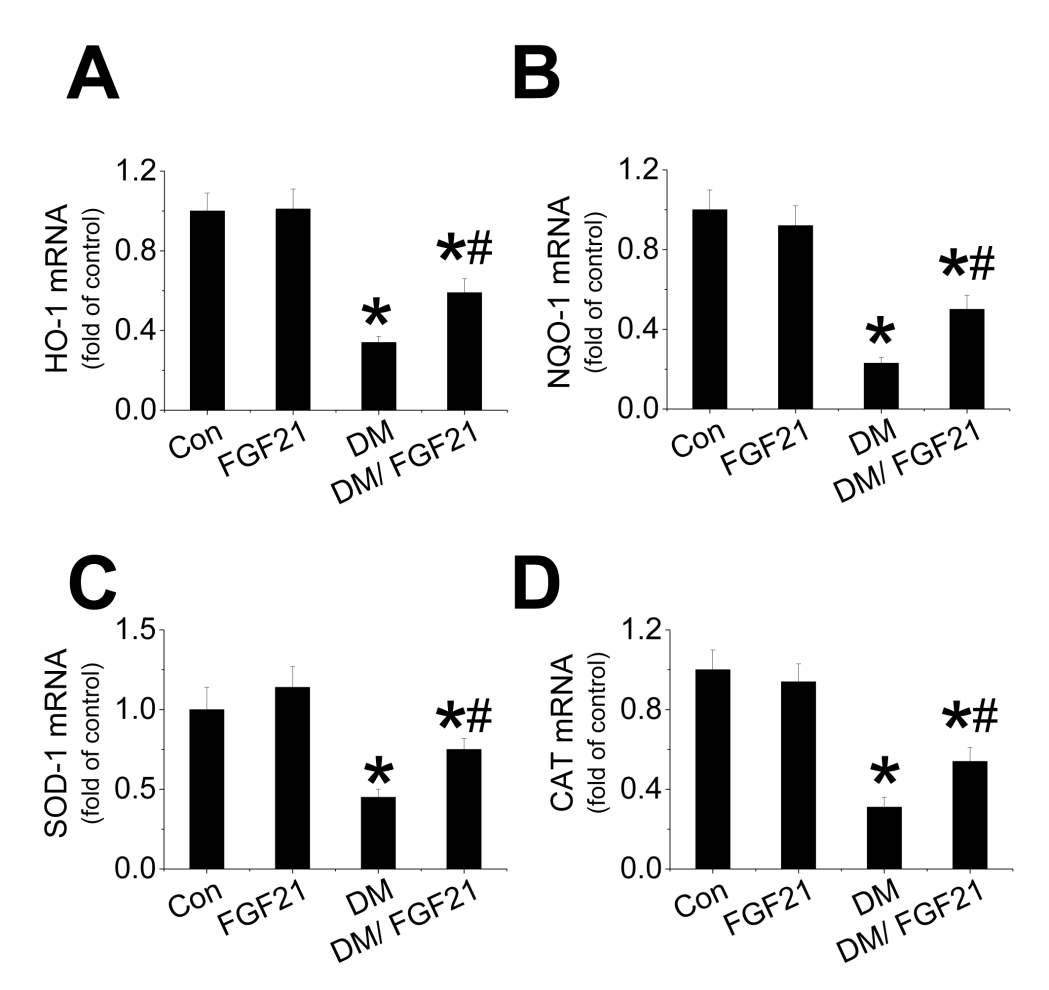
**

**Figure S6. Effect FGF21 supplement on antioxidative genes expression.** The mRNA levels of multiple anti-oxidants including HO-1 (A), NQO-1 (B), CAT (C), SOD-1 (D) were detected with real-time PCR. Data are presented as means ± SD, n = 8/ group. **P* < 0.05 *vs*. the Con group; ^#^*P* < 0.05 *vs.* the DM group.

**
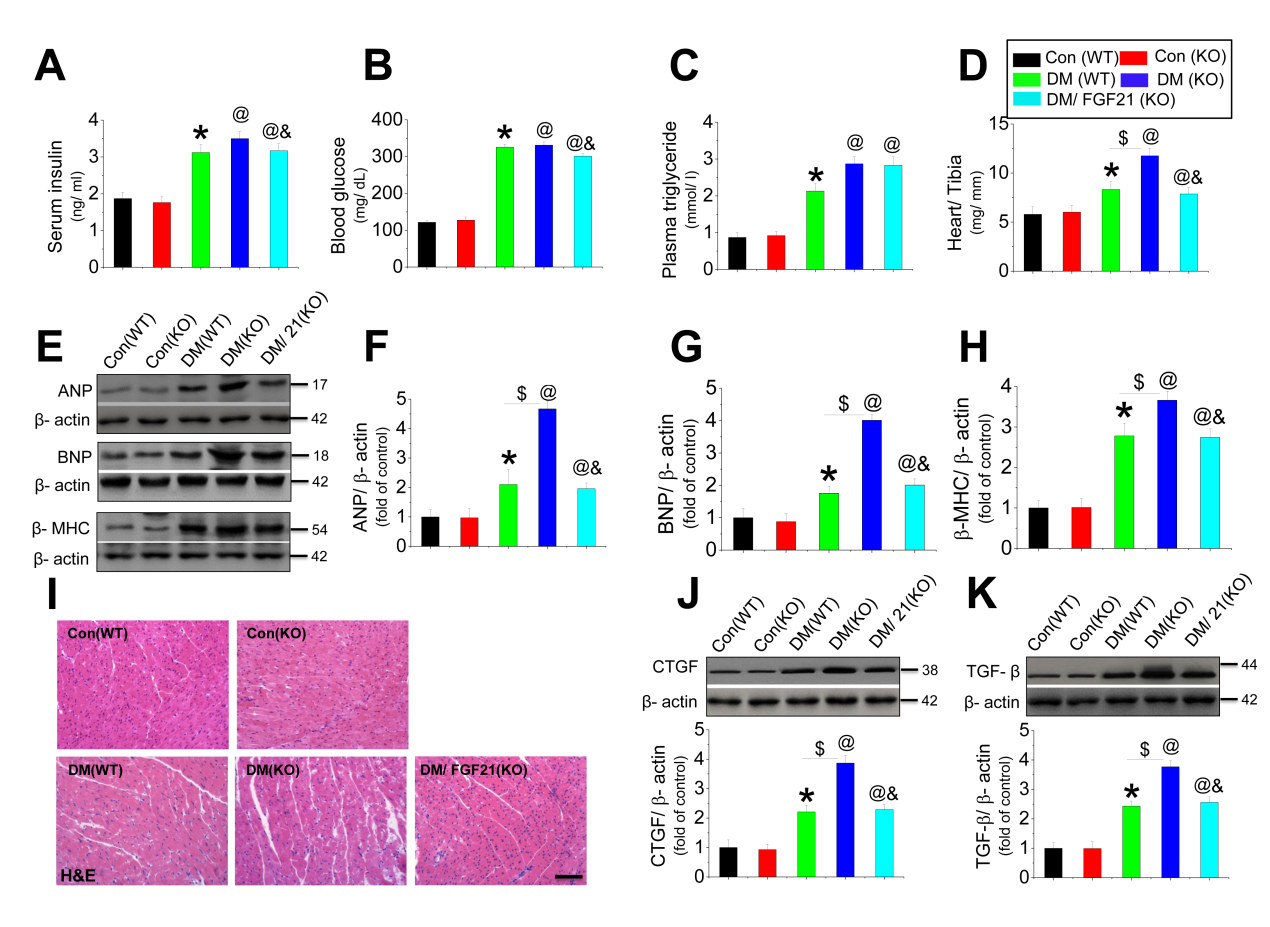
**

**Figure S7. Effect of FGF21 supplement on type 2 diabetes-induced cardiac hypertrophy, histological abnormalities and fibrosis.** Both WT and FGF21-KO mice received HFD/ STZ to induce type 2 diabetes, followed by FGF21 treatment for 4 months. Serum insulin (A), blood glucose (B) and plasma triglyceride (C) were examined by ELISA. Cardiac hypertrophy was evaluated by examining the ratio of heart weight to tibia length (HW/BW, D) and the expression of hypertrophic markers including cardiac ANP (E&F), BNP (E&G), and β-MHC (E&H). Myocardium structure was examined by H&E staining (I). Fibrosis was evaluated by measuring the expression of fibrotic markers such as CTGF (J) and TGF-β (K) in diabetic hearts by Western-blot assay. Data are presented as means ± SD, n = 8/ group. **P* < 0.05 *vs*. the Con group of WT mice; ^@^*P* < 0.05 *vs*. the Con group of FGF21-KO mice; ^$^*P* < 0.05 *vs.* DM group of WT mice; ^&^*P* < 0.05 *vs.* DM group of FGF21-KO mice.

**
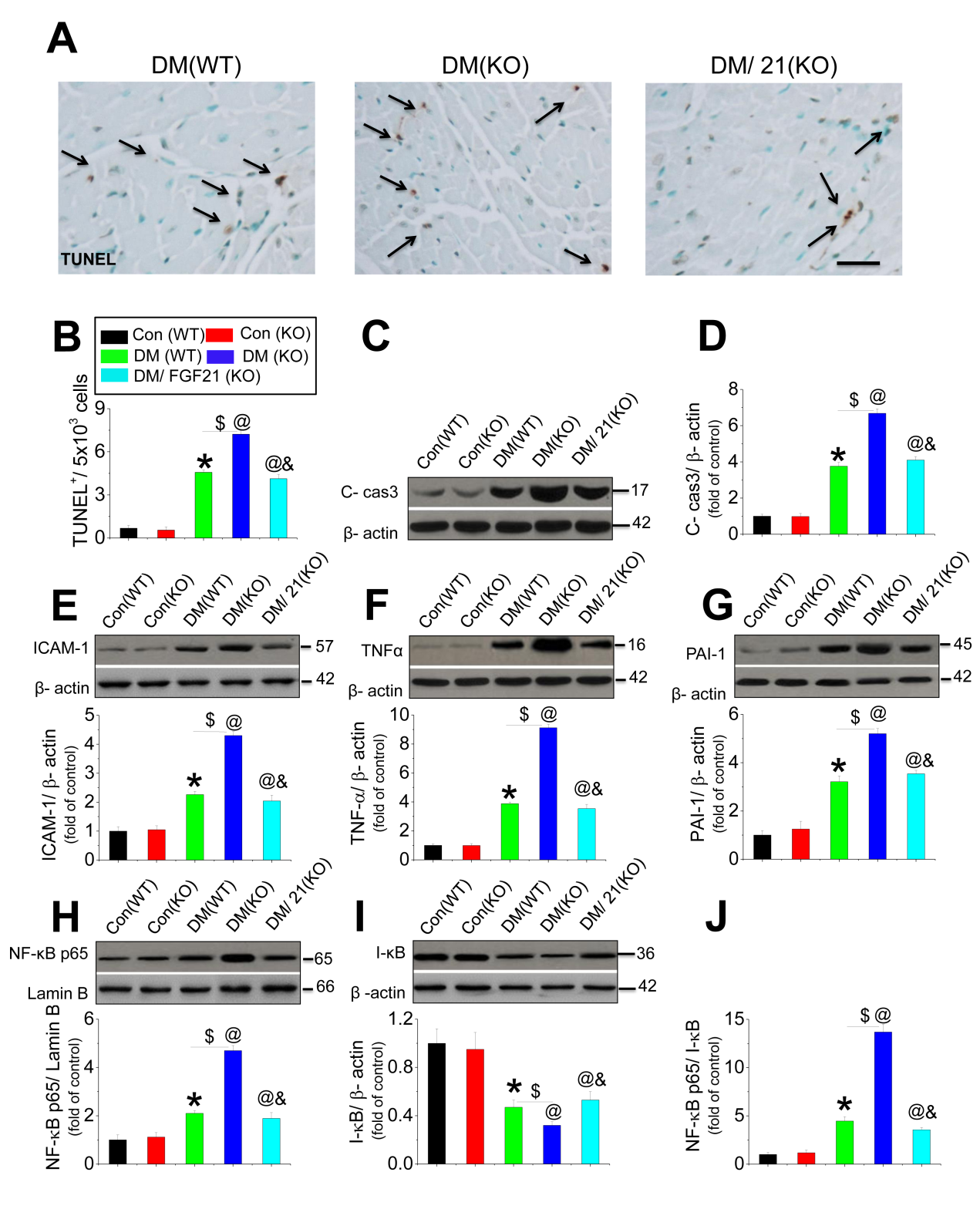
**

**Figure S8. Effect of FGF21 supplement on type 2 diabetes-induced cardiac apoptosis and inflammation.** Apoptosis was measured by TUNEL staining (A), followed by quantitative analysis of TUNEL-positive cells (B). Then cardiac apoptosis was further confirmed by measuring cleaved-caspase-3 expression (C&D) by Western-blot assay. Expressions of inflammatory factors including ICAM-1 (E), TNF-α (F) and PAI-1 (G). Expressions of NF-κB p65 (H), IκB (I), and their ratio (J) were examined by Western blot. Data are presented as means ± SD, n = 8/ group. **P* < 0.05 *vs*. the Con group of WT mice; ^@^*P* < 0.05 *vs*. the Con group of FGF21-KO mice; ^$^*P* < 0.05 *vs.* DM group of WT mice; ^&^*P* < 0.05 *vs.* DM group of FGF21-KO mice.

**
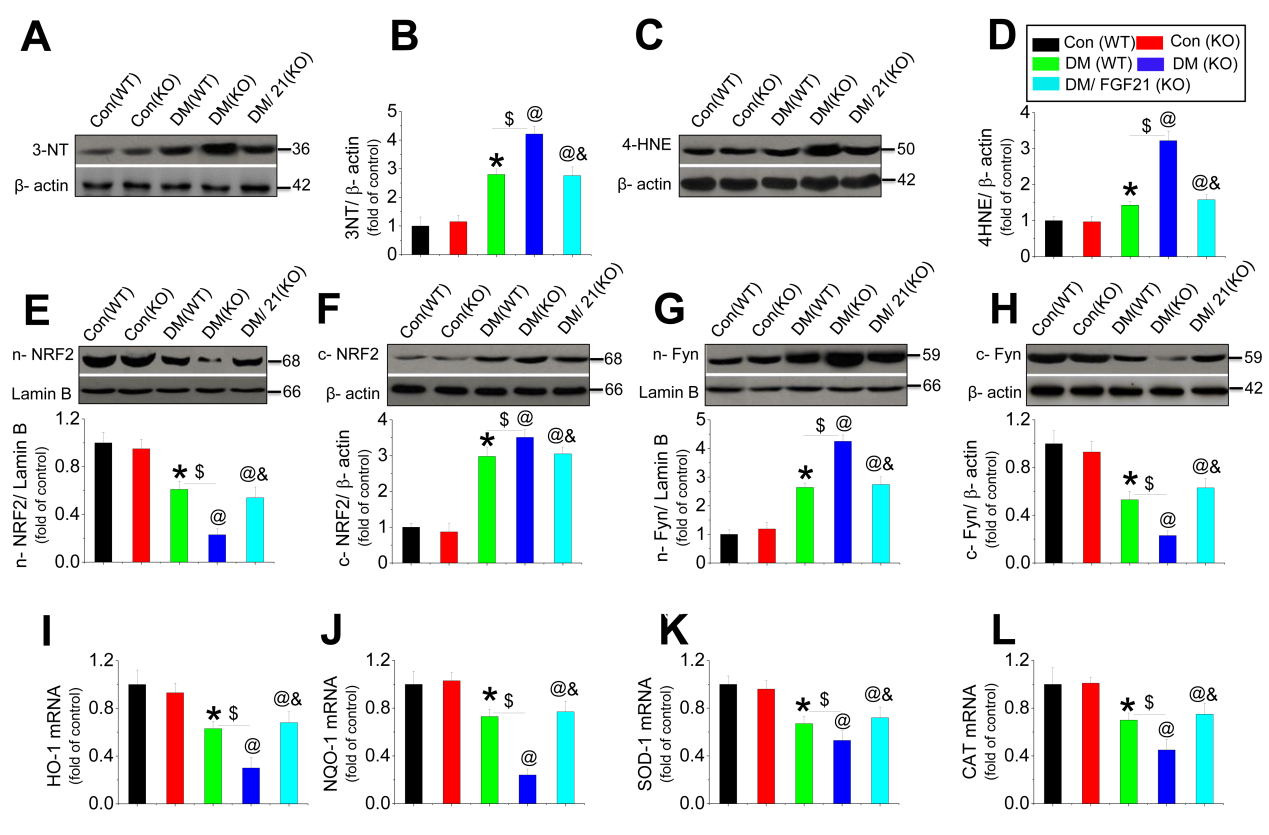
**

**Figure S9. Effect of exogenous FGF21 on diabetes-induced oxidative stress in the diabetic hearts.** The expressions of 3-NT (A&B) and the 4-HNE (C&D) were measured by Western-blot assay. The translocation between the nuclei and cytosol of Nrf2 (E&F) or Fyn (G&H) was evaluated by measuring protein of each in the nuclei and cytosol, respectively. The mRNA levels of multiple anti-oxidants including HO-1 (I), NQO-1 (J), SOD-1 (K), CAT (L) were detected with real-time PCR. Data are presented as means ± SD, n = 8/ group. **P* < 0.05 *vs*. the Con group of WT mice; ^@^*P* < 0.05 *vs*. the Con group of FGF21-KO mice; ^$^*P* < 0.05 *vs.* DM group of WT mice; ^&^*P* < 0.05 *vs.* DM group of FGF21-KO mice.

**
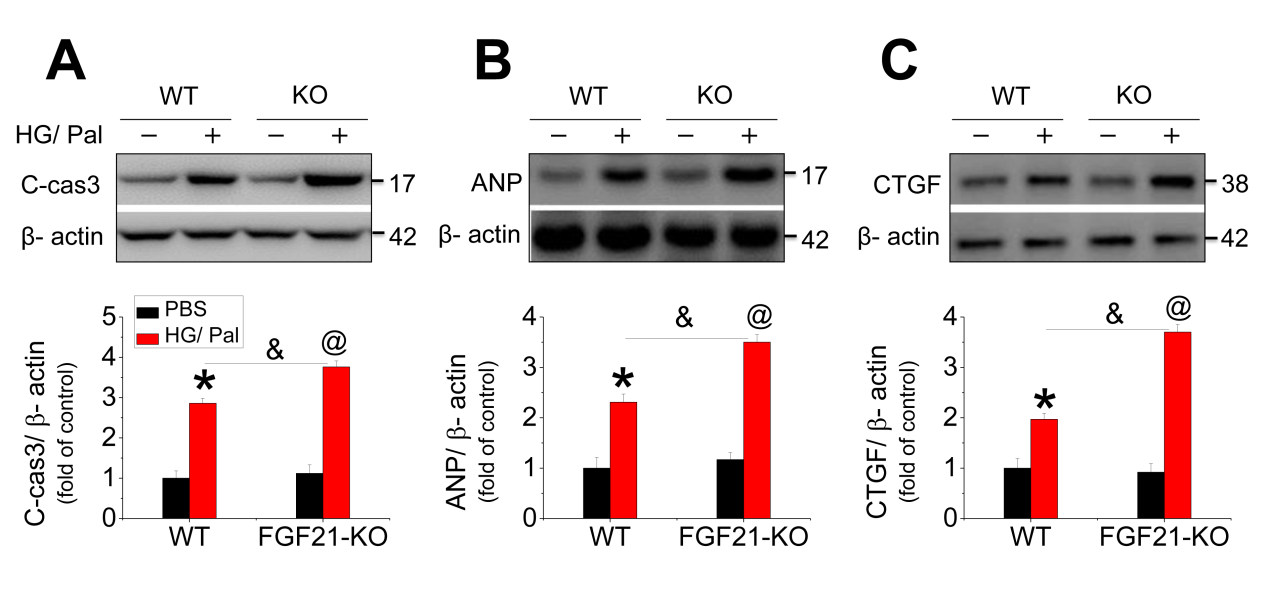
**

**Figure S10. FGF21 deficiency enhanced HG/ Pal-induced cardiomyocytes damage.** Primary cardiomyocytes were isolated form both WT and FGF21-KO neonatal mice and treated both HG/ Pal for 24 hours. The expressions of cleaved-caspase-3 (A), ANP (B) and CTGF (C) were examined by Western-blot assay. Data were collected from at least three independent experiments and presented as mean ± SD. **P* < 0.05 vs PBS-treated group (WT); ^@^*P* < 0.05 vs PBS-treated group (KO); ^&^*P* < 0.05 vs HG/ Pal-treated group (WT).


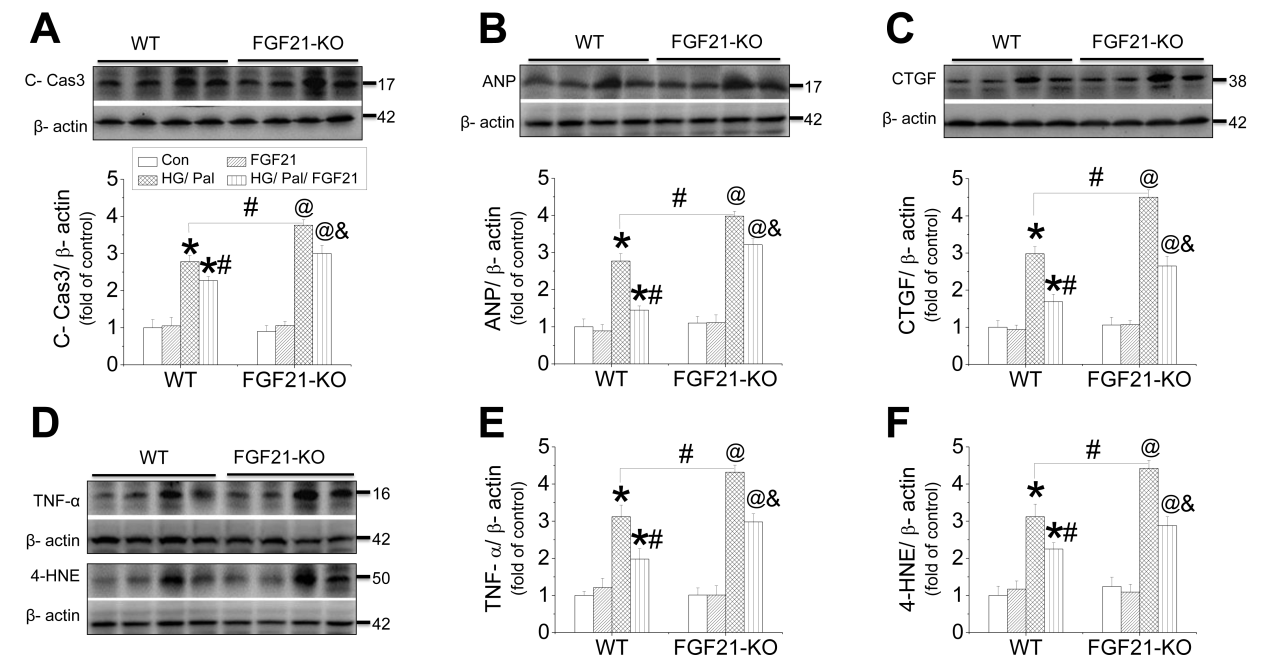


**Figure S11. FGF21 induces protective effect in adult cardiomyocytes against HG/ Pal.** Primary cardiomyocytes was isolated form both Adult WT and FGF21-KO mice and treated both HG/ Pal for 24 hours. Western-blot assay was used to examine the expression of C-caspase-3 (A), ANP (B), CTGF (C), TNF-α (D&E) and 4-HNE (D&F). Data were collected from at least three independent experiments and presented as mean ± SD. **P* < 0.05 vs Con group (WT); ^#^*P* < 0.05 vs HG/ Pal-treated group (WT).^@^*P* < 0.05 vs HG/ Con group (KO); ^&^*P* < 0.05 vs HG/ Pal-treated group (KO).

**
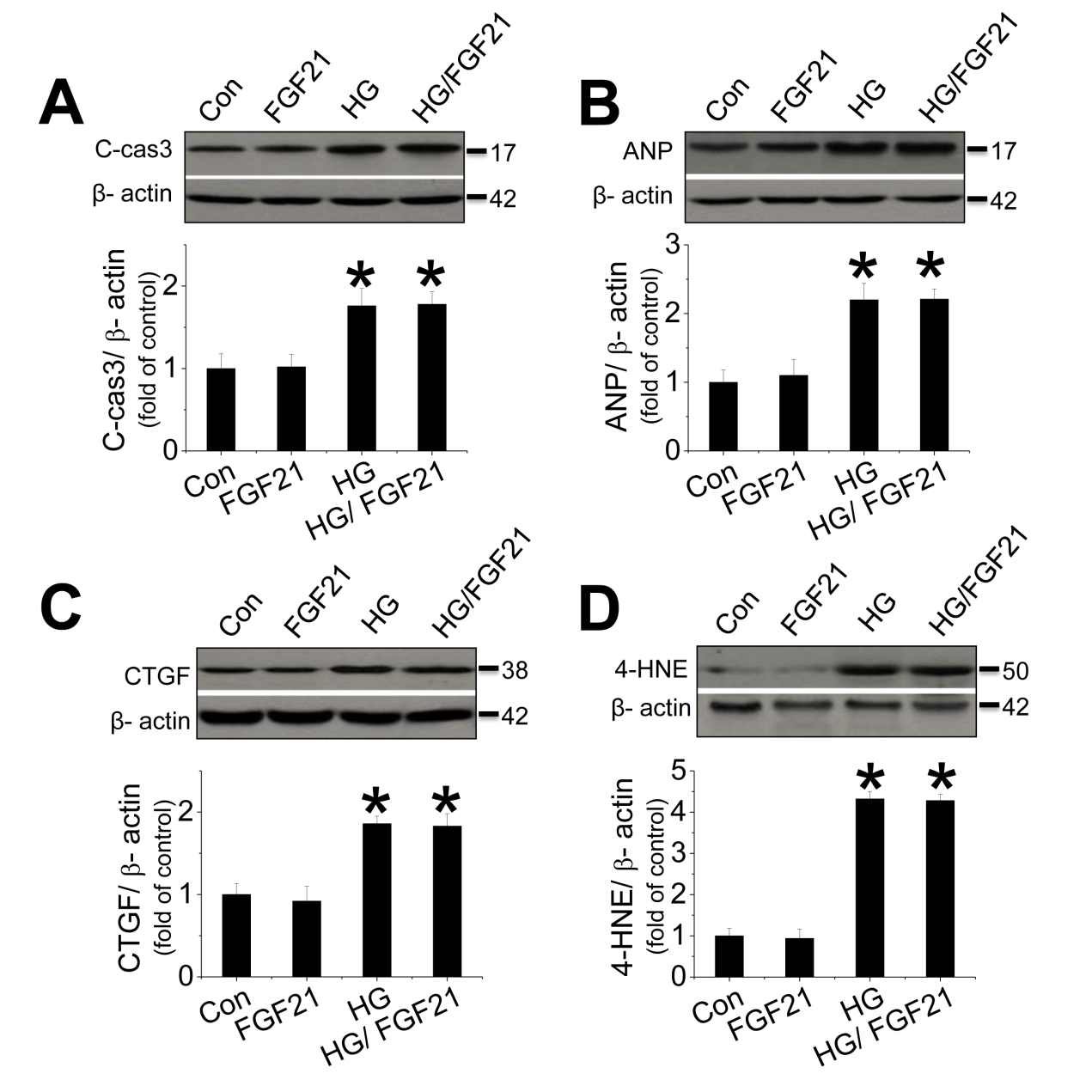
**

**Figure S12. The effect of FGF21 supplement on HG-induced adult mice cardiomyocytes damage.** Primary cardiomyocytes was isolated form both Adult WT and FGF21-KO mice and treated both HG/ Pal for 24 hours. Western-blot assay was used to examine the expression of C-caspase-3 (A), ANP (B), CTGF (C) and 4-HNE (D). Data were collected from at least three independent experiments and presented as mean ± SD. **P* < 0.05 vs Con group.

**
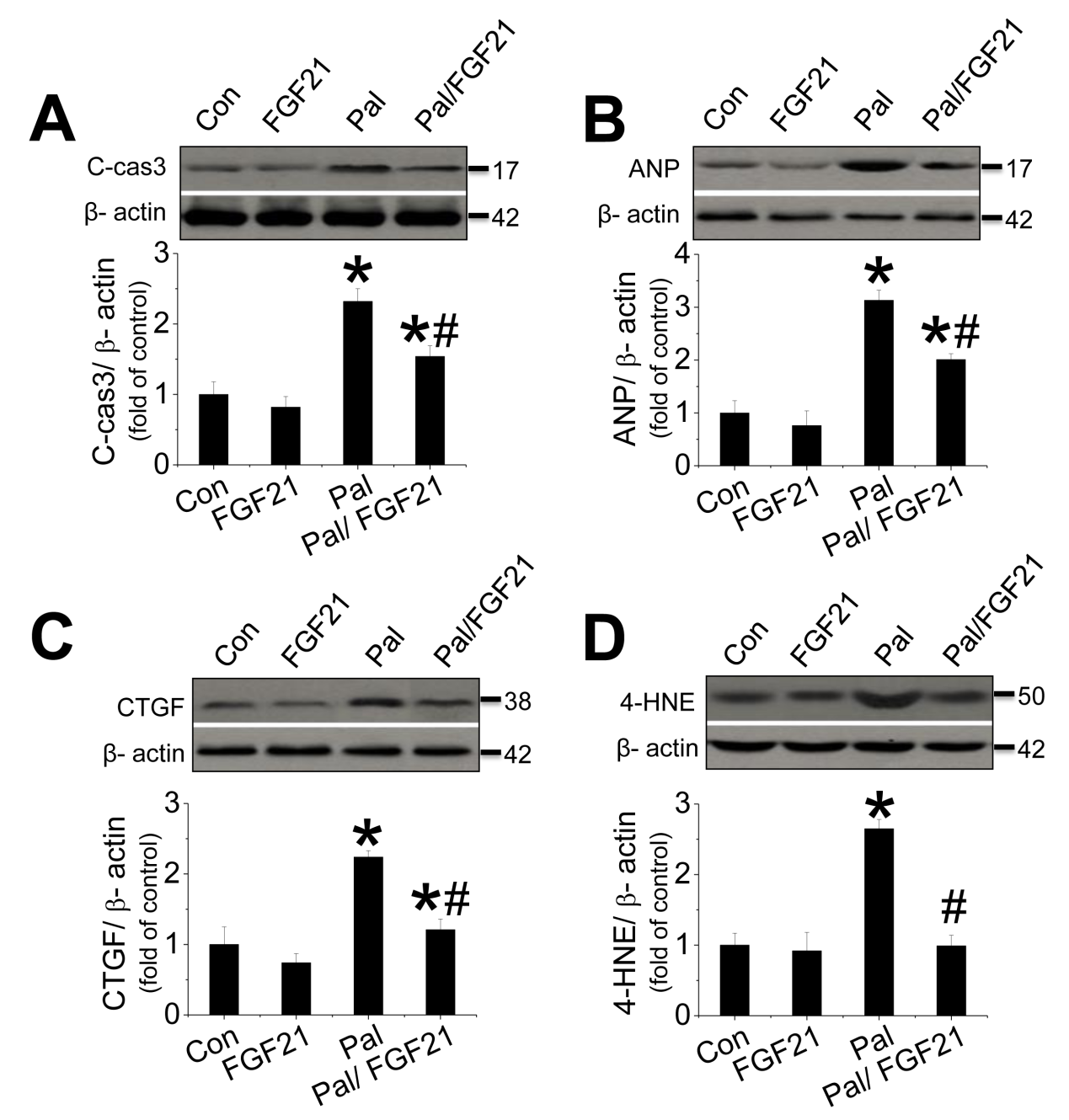
**

**Figure S13. The effect of FGF21 supplement on Pal-induced adult mice cardiomyocytes damage.** Primary cardiomyocytes was isolated form both Adult WT and FGF21-KO mice. Western-blot assay was used to examine the expression of C-caspase-3 (A), ANP (B), CTGF (C) and 4-HNE (D). Data were collected from at least three independent experiments and presented as mean ± SD. **P* < 0.05 vs Con group.

**
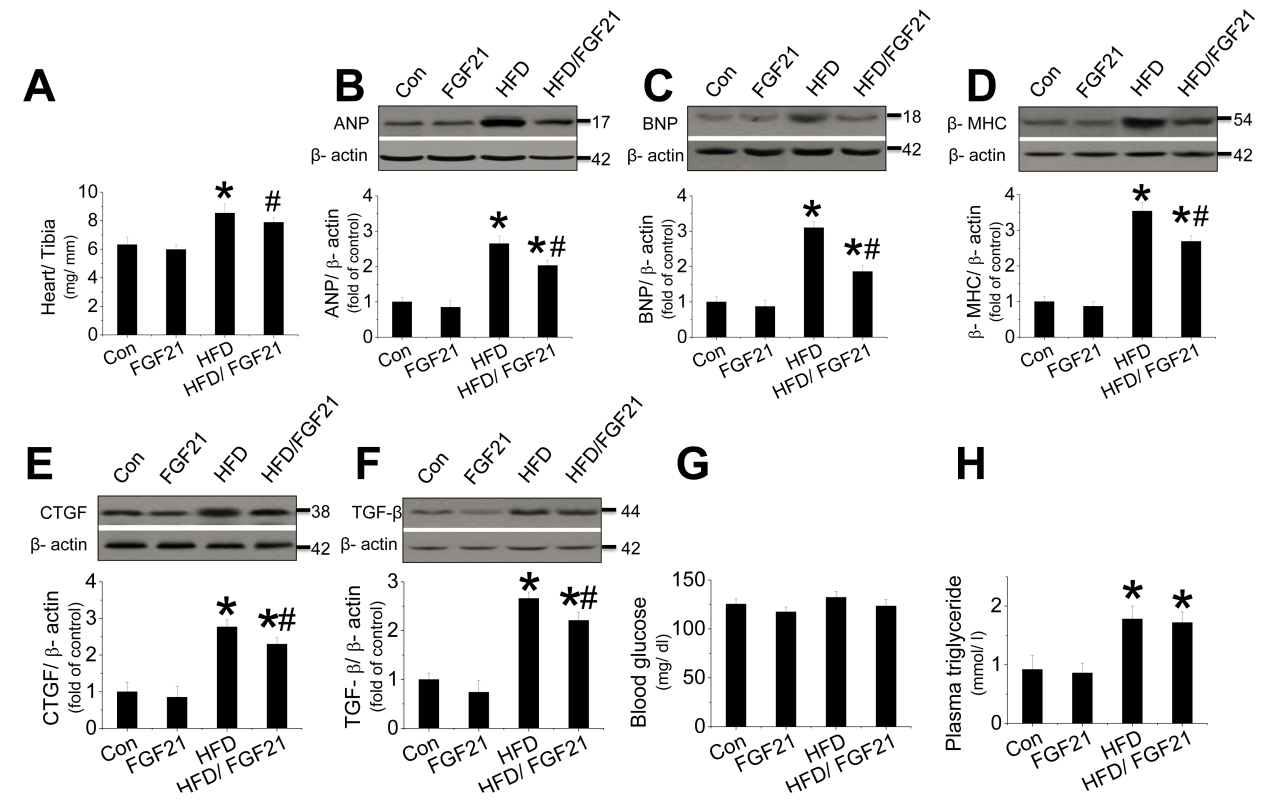
**

**Figure S14. Effect of FGF21 supplement on DIO-induced cardiomyopathy.** HFD -induced DIO mice received FGF21 treatment for 4 months. Then mice were sacrificed and the hearts were isolated. Cardiac hypertrophy was evaluated by examining the ratio of heart weight to tibia length (HW/BW, A) and the expression of hypertrophic markers including cardiac ANP (B), BNP (C), and β-MHC (D). Fibrosis was evaluated by measuring the expressions of cardiac CTGF (E) and TGF-β (F). Addtionally blood glucose level (G) and plasma triglyceride (H) were examined by ELISA. Data are presented as means ± SD, n = 8/ group. **P* < 0.05 *vs*. the control (Con) group; ^#^*P* < 0.05 *vs.* diabetic (DM) group.

**
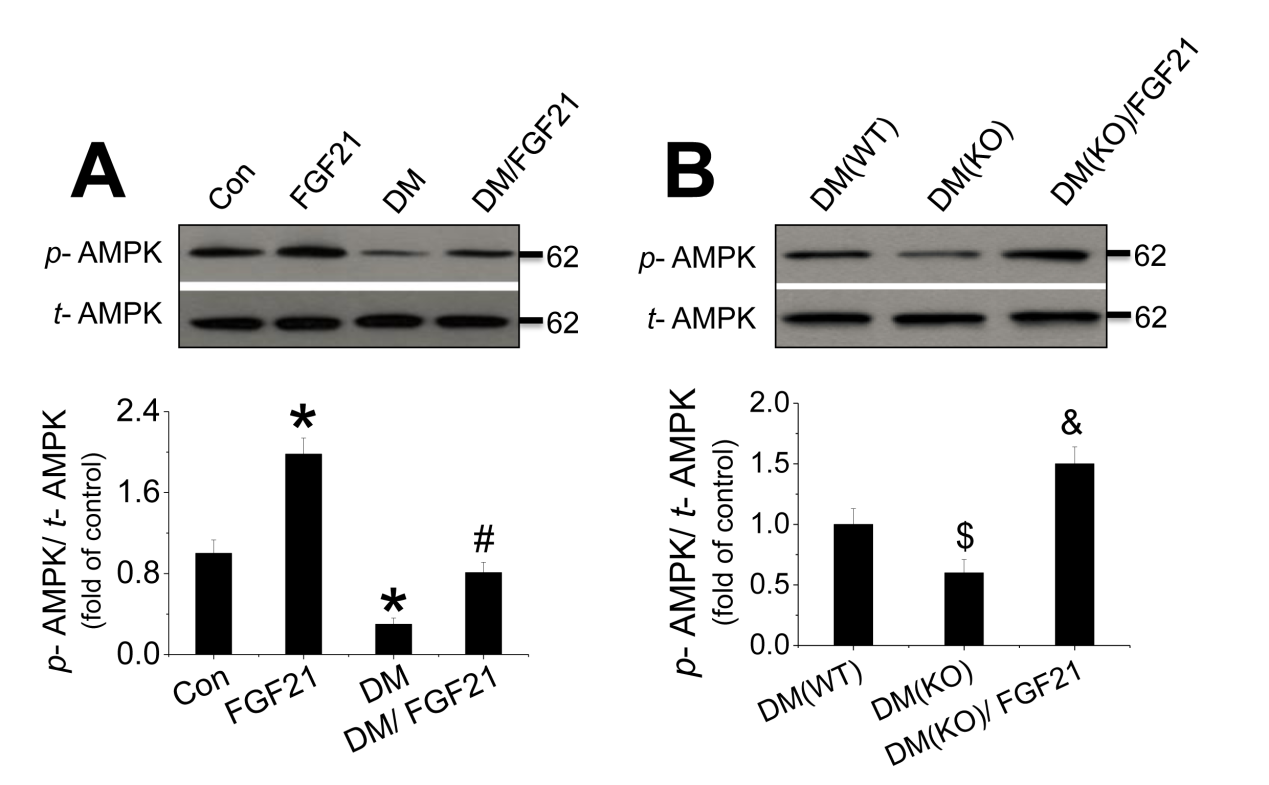
**

**Figure S15. The effect of exogenous and endogenous FGF21 on cardiac AMPK phosphorylation in diabetic mice .** Cardiac tissue from each group of both WT and FGF21-KO mice was used to detected the activity (phosphorylation) of AMPK by Western blot (A&B). Data are presented as means ± SD, n = 8/ group. **P* < 0.05 *vs*. the Con group of WT mice; ^@^*P* < 0.05 *vs*. the Con group of FGF21-KO mice; ^$^*P* < 0.05 *vs.* DM group of WT mice; ^&^*P* < 0.05 *vs.* DM group of FGF21-KO mice.

**
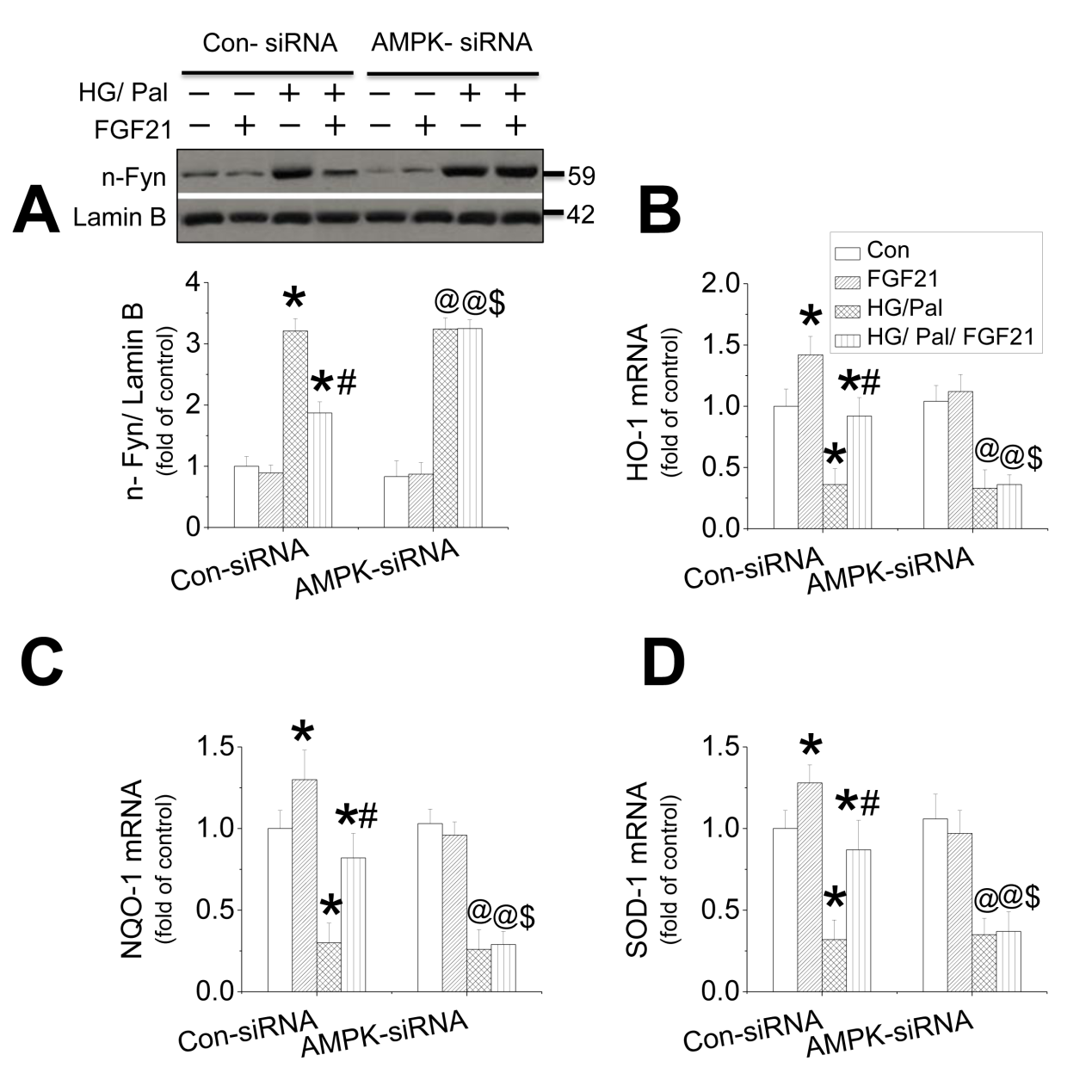
**

**Figure S16. The role of AMPK in FGF21-induced nuclear Fyn reduction and NRF2 downstream antioxidative genes expressions.** Primary cardiomyocytes were isolated and treated with either control or AMPK-specific siRNA and then the cells were co-treating with both HG/ Pal and FGF21 for 24 hours. Western blotting was used to detect the content of nuclear Fyn (A). Additionally, NRF2 downstream genes at the mRNA level including HO-1 (B), NQO-1 (C), SOD-1 (D) were examined by real-time PCR. Data were collected from at least three independent experiments and presented as mean ± SD. **P* < 0.05 vs control in the Con-siRNA group; ^#^*P* < 0.05 vs HG/ Pal in the Con-siRNA group; ^@^*P* < 0.05 vs control in AMPK-siRNA group; ^$^*P* < 0.05 vs HG/ Pal/ FGF21 in the Con-siRNA group.

**
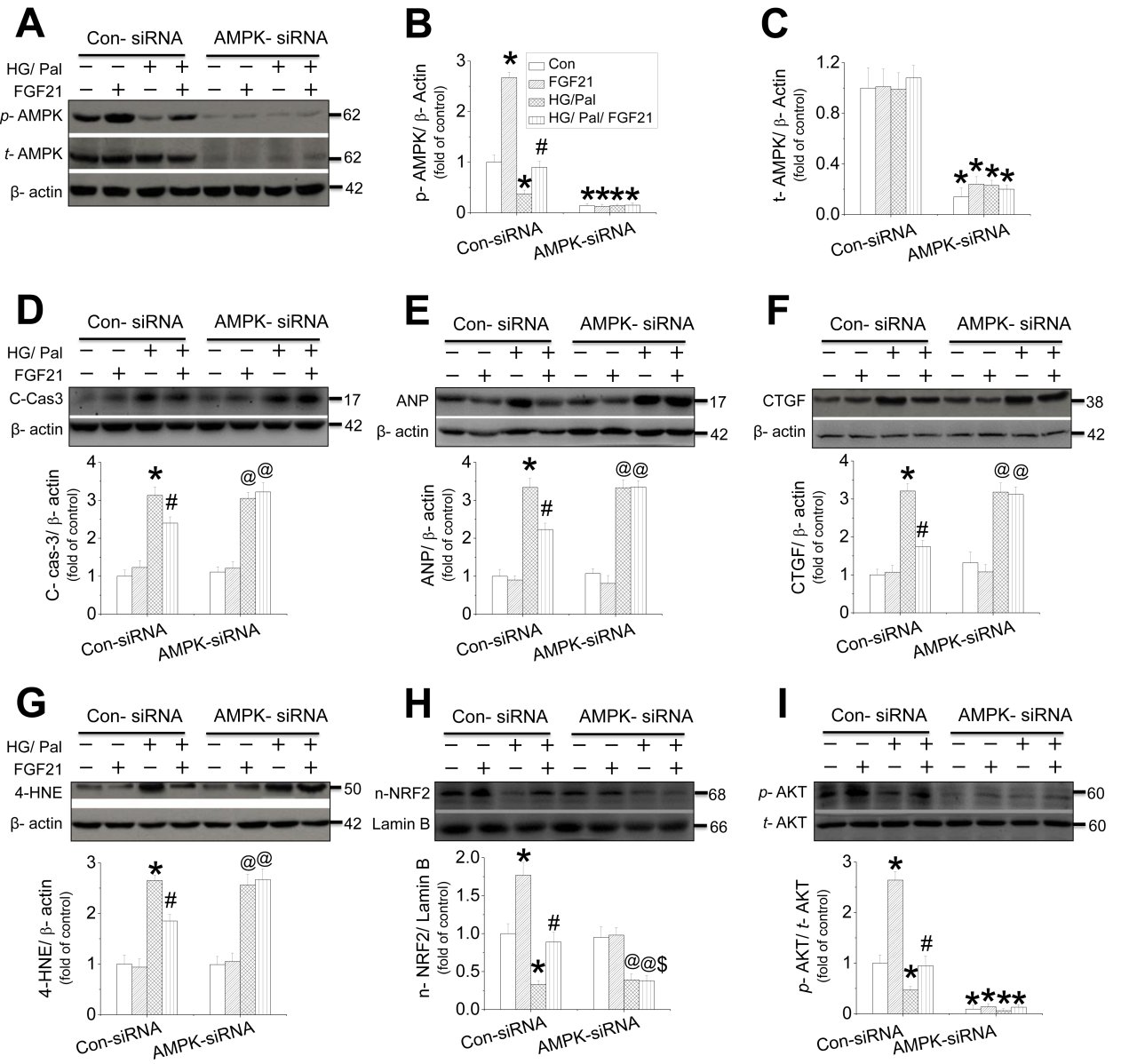
**

**Figure S17. The role of AMPK in FGF21-induced protection in adult mouse cardiomyocytes against HG/ Pal.** Primary cardiomyocytes were isolated from adult mice and treated with either control or AMPK-specific siRNA and then the cells were co-treating with both HG/ Pal and FGF21 for 24 hours. Western blotting was used to detect the phosphorylation (A&B) and expression levels of AMPK (A&C). Under this circumstance, the expressions of cleaved-caspase 3 (D), ANP (E), CTGF (F) and 4-HNE (G) were measured by Western-blot assay. The nuclear accumulation of NRF2 (H) and AKT phosphorylation (I) were evaluated by Western-blot assay. Data were collected from at least three independent experiments and presented as mean ± SD. **P* < 0.05 vs control in the Con-siRNA group; ^#^*P* < 0.05 vs HG/ Pal in the Con-siRNA group; ^@^*P* < 0.05 vs control in AMPK-siRNA group; ^$^*P* < 0.05 vs HG/ Pal/ FGF21 in the Con-siRNA group.

**
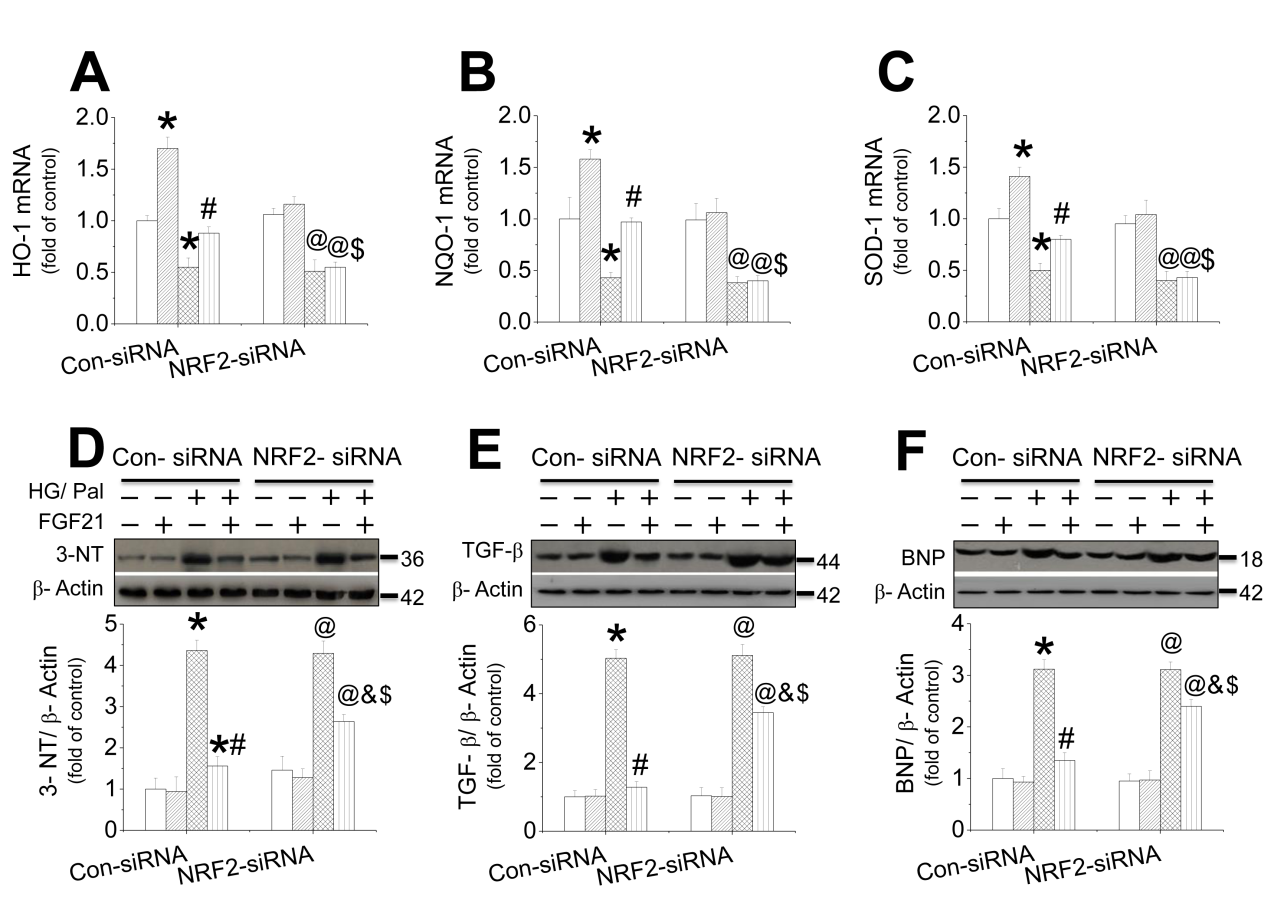
**

**Figure S18. FGF21 prevents HG/ Pal-induced oxidative stress, fibrotic effect and hypertrophy via NRF2 in neonatal mouse cardiomyocytes.** Primary cardiomyocytes were isolated and treated with either control or NRF2-specific siRNA and then the cells were co-treating with both HG/ Pal and FGF21 for 24 hours. The mRNA levels of NRF2 down-stream antioxidants including HO-1 (A), NOQ-1 (B) and SOD-1 (C) were measured by Real-time PCR. The expressions of 3-NT and 4-HNE (D), TGF-β (E), and BNP (F) were measured by Western-blot assay. Data were collected from at least three independent experiments and presented as mean ± SD. **P* < 0.05 vs control in the Con-siRNA group; ^#^*P* < 0.05 vs HG/ Pal in the Con-siRNA group; ^@^*P* < 0.05 vs control in NRF2-siRNA group; ^&^*P* < 0.05 vs HG/ Pal in the NRF2-siRNA group; ^$^*P* < 0.05 vs HG/ Pal/ FGF21 in the Con-siRNA group.

**
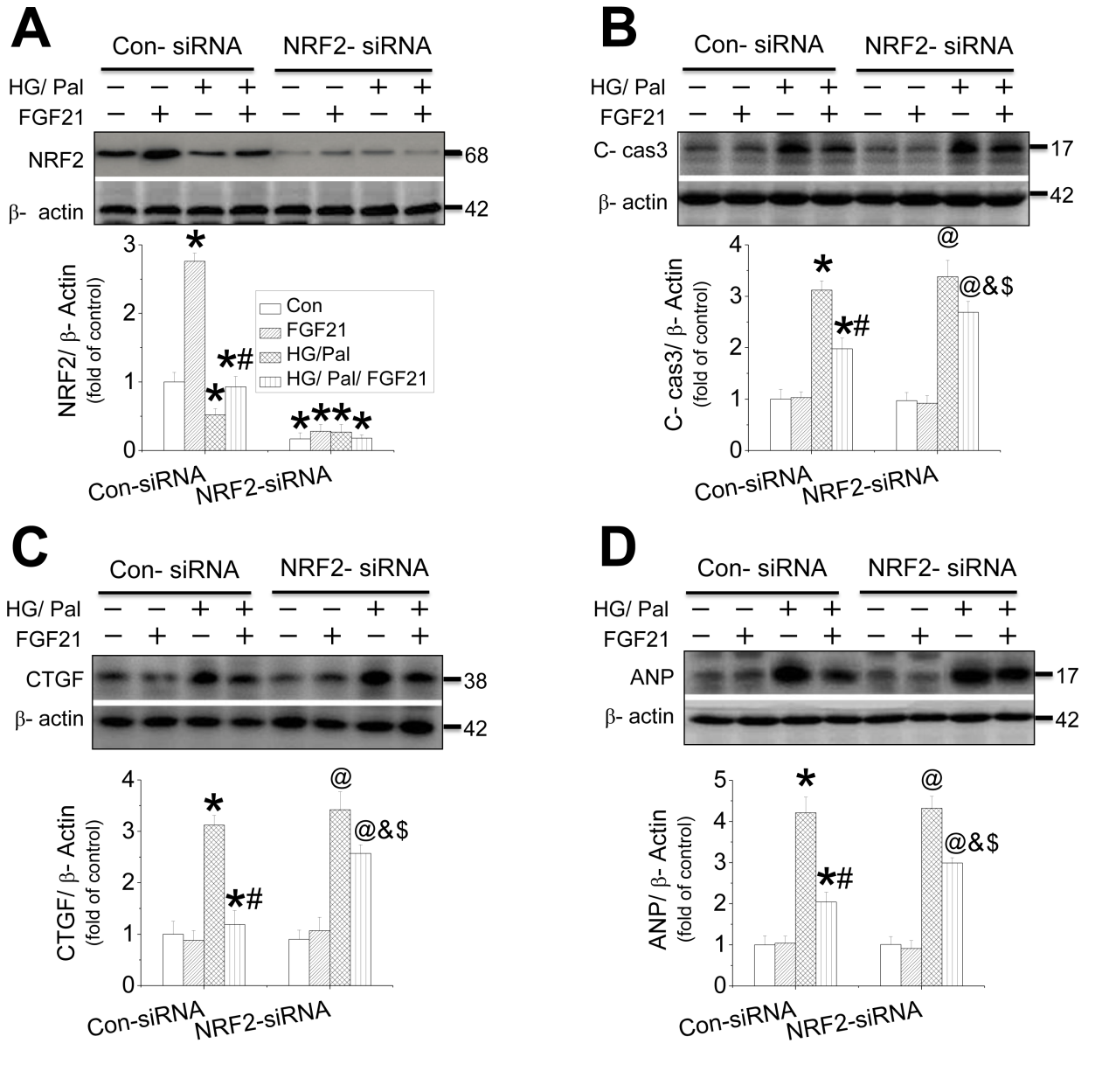
**

**Figure S19. FGF21 prevents HG/ Pal-induced apoptosis, fibrotic effect and hypertrophy via NRF2 in adult mouse cardiomyocytes.** Primary cardiomyocytes were isolated from adult mouse and treated with either control or NRF2-specific siRNA and then the cells were co-treating with both HG/ Pal and FGF21 for 24 hours. The expressions of NRF2 (A), C-caspase-3 (B), CTGF (C) and ANP (D) were measured by Western-blot assay. Data were collected from at least three independent experiments and presented as mean ± SD. **P* < 0.05 vs control in the Con-siRNA group; ^#^*P* < 0.05 vs HG/ Pal in the Con-siRNA group; ^@^*P* < 0.05 vs control in NRF2-siRNA group; ^$^*P* < 0.05 vs HG/ Pal/ FGF21 in the Con-siRNA group.

**
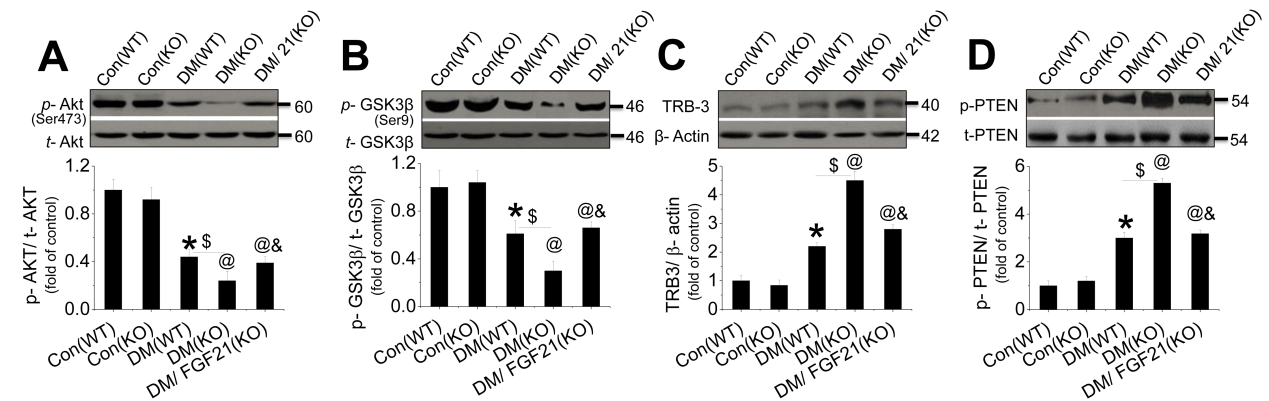
**

**Figure S20. The impacts of FGF21 supplement on AKT signaling in diabetic hearts.** Cardiac tissue from WT and FGF21-KO mice with or without diabetes was used to measure the phosphorylation of AKT (A) and GSK3β (B) as well as the levels of AKT’s negative regulator including TRB3 expression (C) and PTEN phosphorylation (D) by Western-blot assay. Data are presented as means ± SD, n = 8/ group. **P* < 0.05 *vs*. the Con group (WT); ^@^*P* < 0.05 *vs.* the Con group (KO); ^$^*P* < 0.05 *vs*. the DM group (WT); ^$^*P* < 0.05 *vs*. the DM group (KO).

**
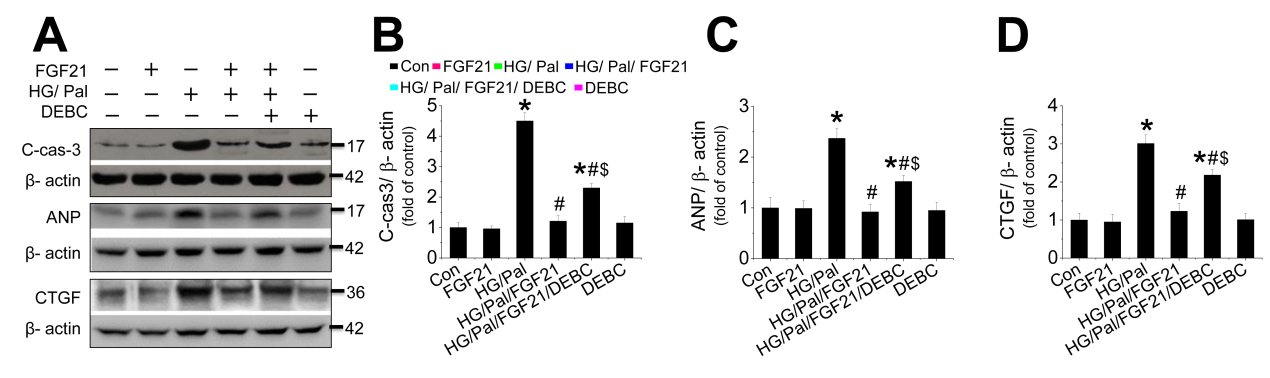
**

**Figure S21. The role AKT activation in FGF21-induced protective effect against HG/ Pal in neonatal mouse cardiomyocytes**. AKT specific inhibitor (DEBC) was used to identify the role of AKT signaling in FGF21 induced cardiomyocytes protection by detecting the expressions of cleaved-caspase-3 (A&B), ANP (A&C) and CTGF (A&D) by Westernblot assay. Data were collected from at least three independent experiments and presented as mean ± SD. **P* < 0.05 vs control; ^#^*P* < 0.05 vs HG/ Pal; ^$^*P* < 0.05 vs HG/ Pal/ FGF21.

**
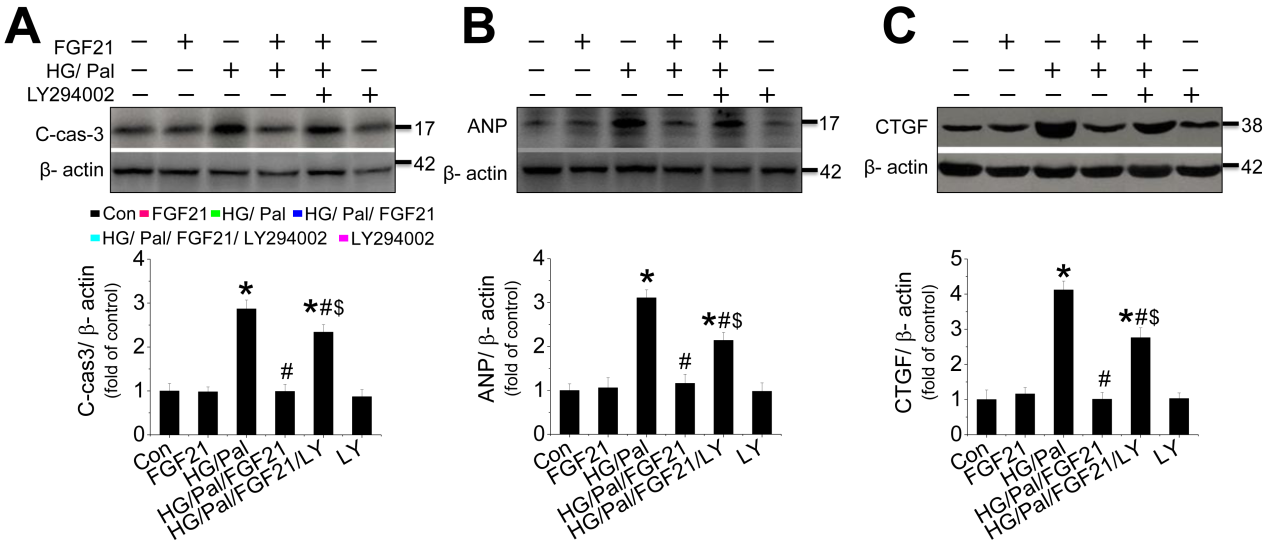
**

**Figure S22. The role AKT activation in FGF21-induced protective effect against HG/ Pal in adult mouse cardiomyocytes**. LY294002 was used to identify the role of AKT signaling in FGF21 induced adult mouse cardiomyocytes protection by detecting the expressions of cleaved-caspase-3 (A), ANP (B) and CTGF (C) by Western-blot assay. Data were collected from at least three independent experiments and presented as mean ± SD. **P* < 0.05 vs control; ^#^*P* < 0.05 vs HG/ Pal; ^$^*P* < 0.05 vs HG/ Pal/ FGF21. LY = LY294002.

**
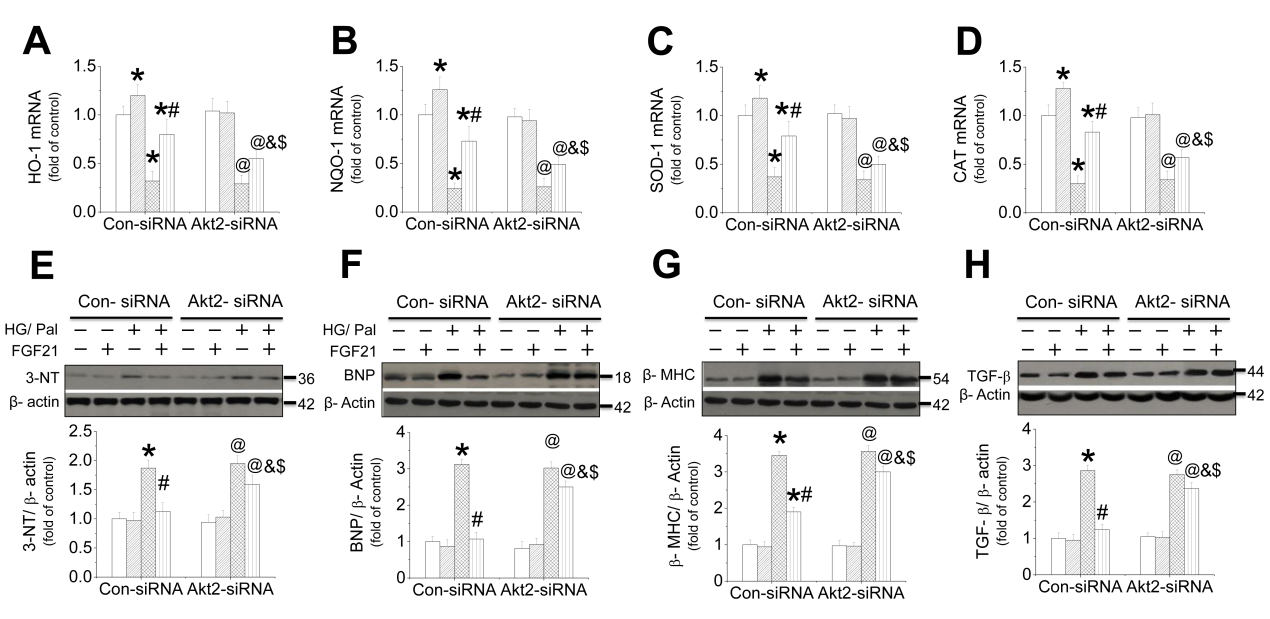
**

**Figure S23. FGF21 prevents HG/ Pal-induced oxidative stress, fibrotic effect and hypertrophy via AKT2 in cardiomyocytes.** Primary cardiomyocytes were isolated and treated with either control or AKT2-specific siRNA and then the cells were co-treating with both HG/ Pal and FGF21 for 24 hours. The mRNA levels of NRF2 down-stream antioxidants including HO-1 (A), NOQ-1 (B), SOD-1 (C) and CAT (D) were measured by Real-time PCR. The expressions of 3-NT and 3-NT (E), BNP (F), β- MHC (G) and TGF-β (H) were measured by Western-blot assay. Data were collected from at least three independent experiments and presented as mean ± SD. **P* < 0.05 vs control in the Con-siRNA group; ^#^*P* < 0.05 vs HG/ Pal in the Con-siRNA group; ^@^*P* < 0.05 vs control in NRF2-siRNA group; ^&^*P* < 0.05 vs HG/ Pal in the NRF2-siRNA group; ^$^*P* < 0.05 vs HG/ Pal/ FGF21 in the Con-siRNA group.

**
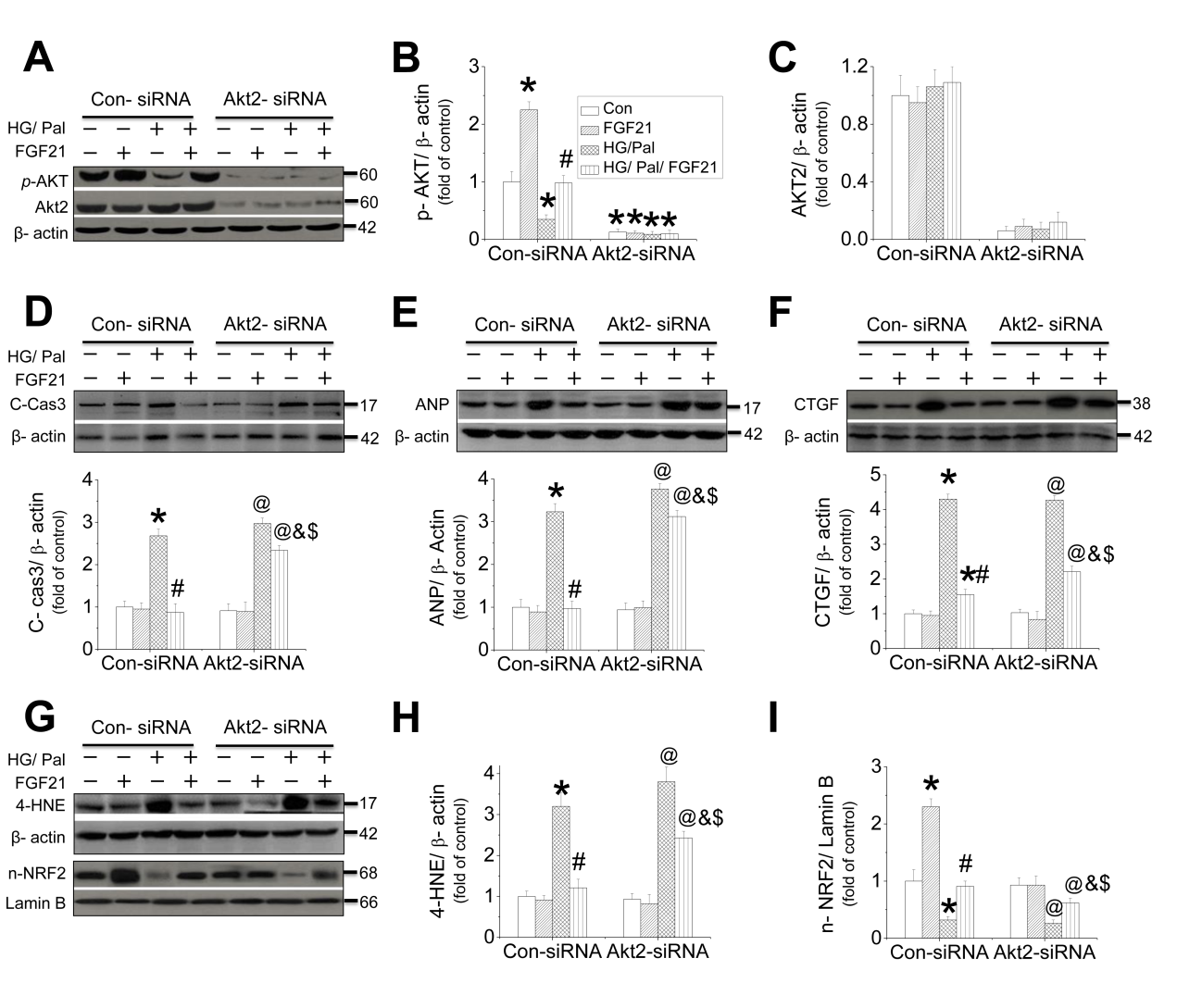
**

**Figure S24. The role of AKT2 in FGF21-induced protection in adult mouse cardiomyocytes against HG/ Pal.** Primary cardiomyocytes were isolated from adult mouse and treated with either control or AKT2-siRNA and then the cells were co-treating with both HG/ Pal and FGF21 for 24 hours. Western blotting was used to detect AKT phosphorylation (A&B) and AKT2 expression (A&C). The expressions of cleaved-caspase 3 (D), ANP (E), CTGF (F), 4-HNE (G&H) and nuclear NRF2 (G&I) were measured by Western-blot assay. Data were collected from at least three independent experiments and presented as mean ± SD. **P* < 0.05 vs control in the Con-siRNA group; ^#^*P* < 0.05 vs HG/ Pal in the Con-siRNA group; ^@^*P* < 0.05 vs control in AKT2-siRNA group; ^&^*P* < 0.05 vs HG/ Pal in the AKT2-siRNA group; ^$^*P* < 0.05 vs HG/ Pal/ FGF21 in the Con-siRNA group.

**
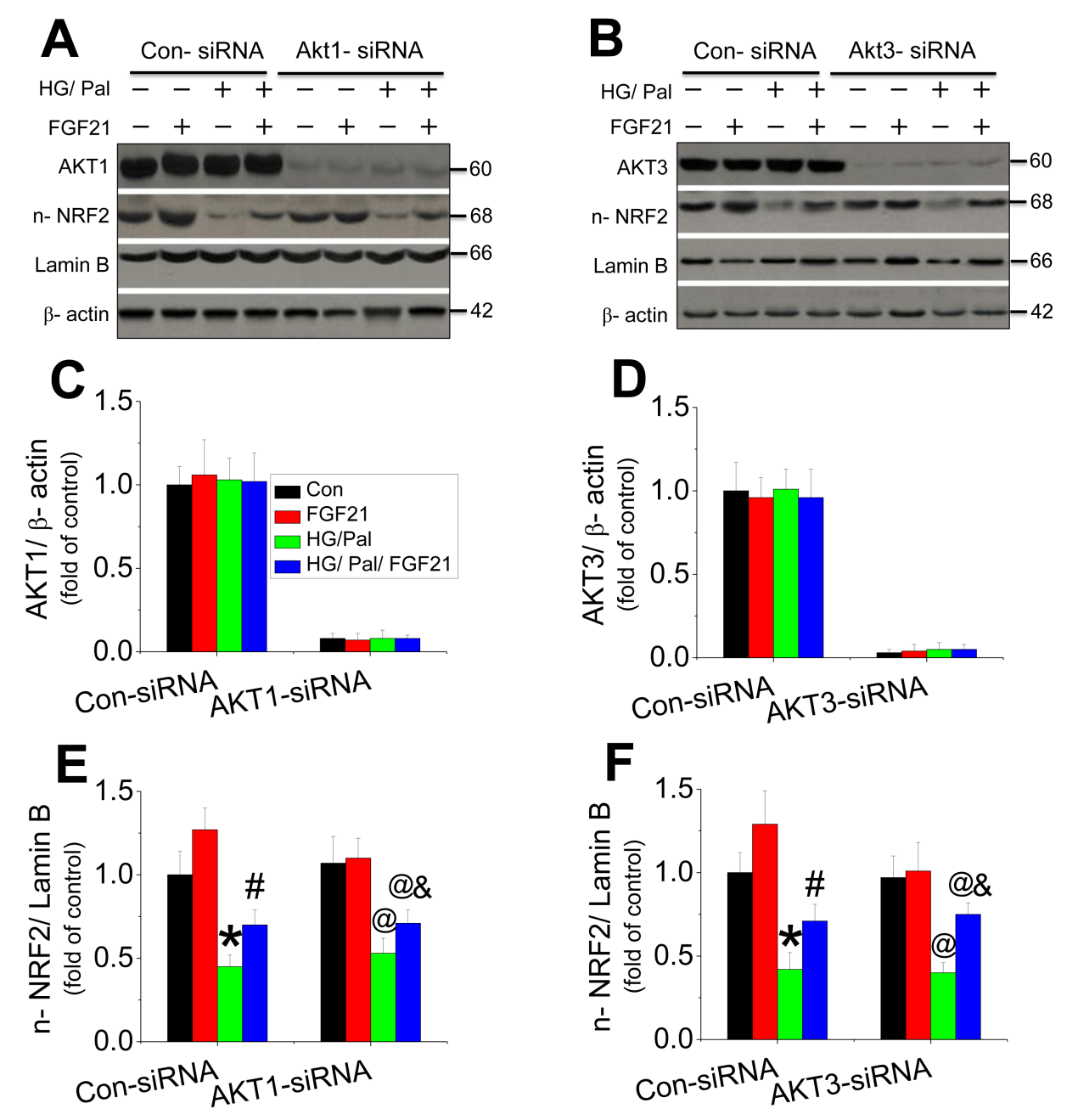
**

**Figure S25. The Impact of AKT1 or 3 depletion on FGF21-induced cardiomyocytes protection against HG/ Pal.** Primary cardiomyocytes were isolated and treated with either control or AKT1-siRNA/ AKT3-siRNA, and then the cells were co-treating with both HG/ Pal and FGF21 for 24 hours. Under the condition of AKT1-siRNA treatment, AKT1 expression (A&C) and nuclear NRF2 level (A&E) were examined by Western blot. Meanwhile, Under the condition of AKT3-siRNA treatment, AKT3 expression (B&D) and nuclear NRF2 level (B&E) were examined by Western blot. Data were collected from at least three independent experiments and presented as mean ± SD. **P* < 0.05 vs control in the Con-siRNA group; ^#^*P* < 0.05 vs HG/ Pal in the Con-siRNA group; ^@^*P* < 0.05 vs control in AKT1 or 3-siRNA group; ^&^*P* < 0.05 vs HG/ Pal in the AKT1 or 3-siRNA group.


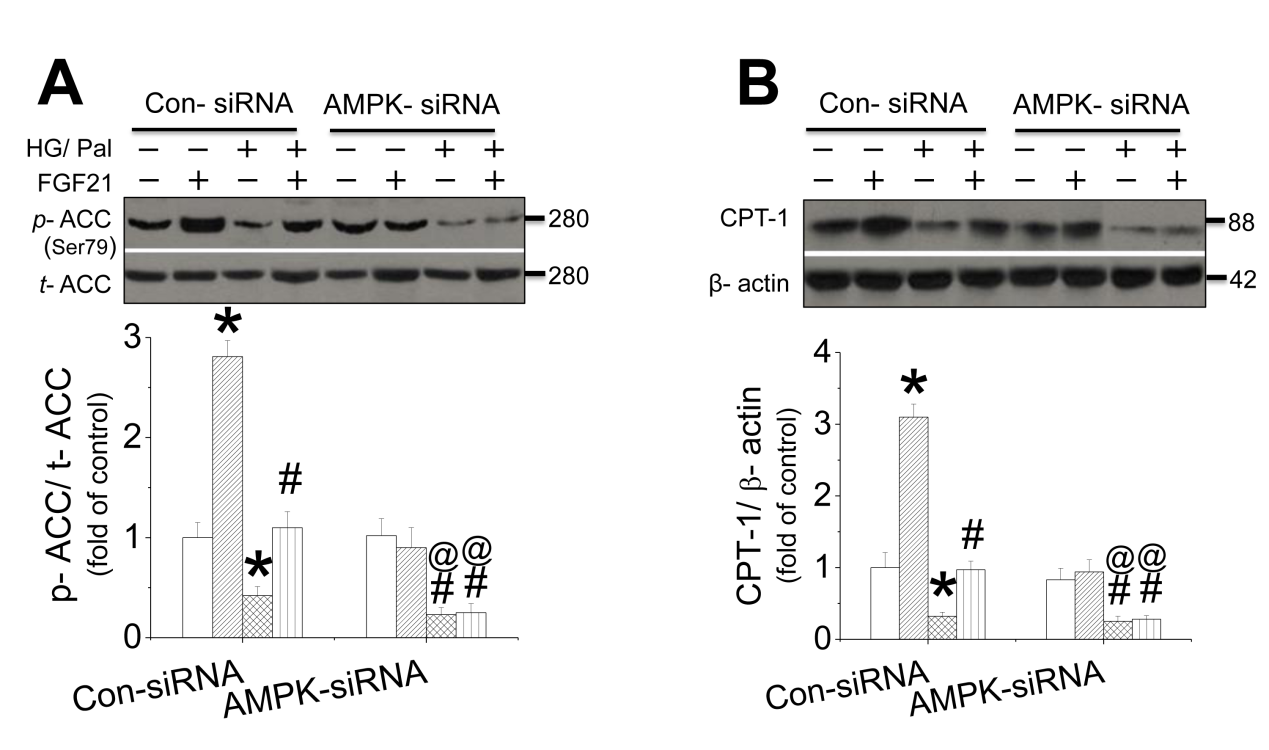


**Figure S26. The role of AMPK in FGF21 induced activation of ACC-CPT-1 pathway in adult cardiomyocytes.** Primary cardiomyocytes were isolated form adult mouse and treated with either control or AMPK-siRNA, and then the cells were co-treating with both HG/ Pal and FGF21 for 24 hours. ACC phosphorylation (A) and CPT-1 expression (B) were determined by Western-blot assay. Data were collected from at least three independent experiments and presented as mean ± SD. **P* < 0.05 vs control in the Con-siRNA group; ^#^*P* < 0.05 vs HG/ Pal in the Con-siRNA group; ^@^*P* < 0.05 vs control in the AMPK-siRNA group.


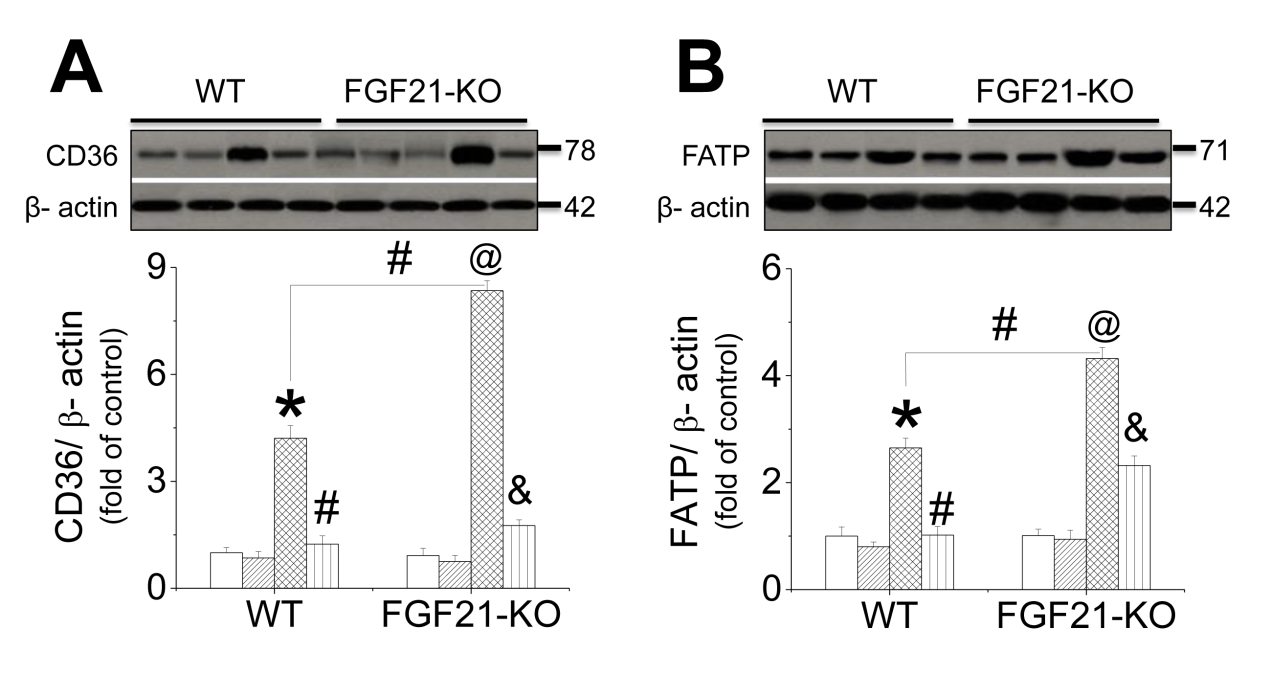


**Figure S27. The effect of FGF21 on cardiac lipid absorption.** The expressions of CD36 (A) and FATP (B), marker of lipid cellular absorption, in the hearts of both WT and FGF21-KO mice were examined by Western blot. Data are presented as means ± SD, n = 8/ group. **P* < 0.05 *vs*. the Con group in WT mice; ^#^*P* < 0.05 *vs.* the DM group in WT mice; ^@^*P* < 0.05 *vs*. the Con group in FGF21-KO mice; ^&^*P* < 0.05 *vs.* the DM group in FGF21-KO mice.


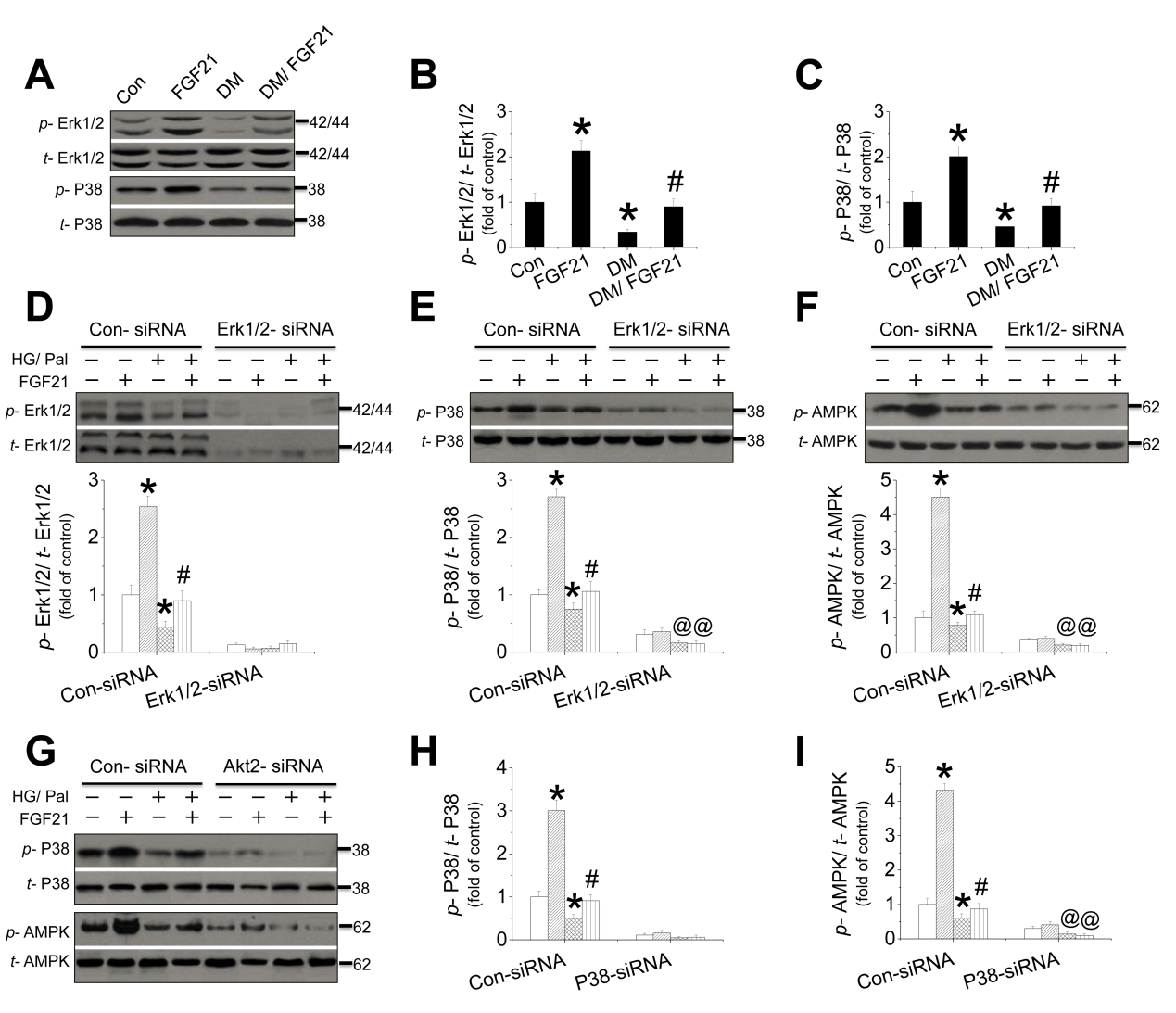


**Figure S28. The role of Erk1/2-p38 MAPK pathway in FGF21-induced activation of AMPK in diabetic hearts and HG/ Pal-treated neonatal mouse cardiomyocytes.** HFD/ STZ-induced type 2 diabetic and age-matched mice received FGF21 treatment for 4 months. Then mice were sacrificed and the hearts were isolated. The phosphorylation of both cardiac Erk1/2 (A&B) and p38 MAPK (A&C) were examined by Western-blot assay. Addtionally, primary cardiomyocytes were isolated form neonatal mouse and treated with either Erk1/2-siRNA or p38 MAPK-siRNA, and then the cells were co-treating with both HG/ Pal and FGF21 for 24 hours. The Erk1/2 (D), p38 MAPK (E) and AMPK (F) phosphorylation in the cardiomyocytes pre-treated with Erk1/2-siRNA were examined by Western-blot assay. Meanwhile p38 MAPK (G&H) and AMPK (G&I) phosphorylation in the cardiomyocytes pre-treated with p38 MAPK-siRNA were also examined by Western-blot assay. For in vivo study (A-C), data are presented as means ± SD, n = 8/ group. **P* < 0.05 *vs*. the Con group; ^#^*P* < 0.05 *vs.* the DM group. For in vitro study, data were collected from at least three independent experiments and presented as mean ± SD. **P* < 0.05 vs control in the Con-siRNA group; ^#^*P* < 0.05 vs HG/ Pal in the Con-siRNA group; ^@^*P* < 0.05 vs control in the AMPK-siRNA or p38 MAPK-siRNA group.
